# The Hydrophobic Effects: Our Current Understanding

**DOI:** 10.3390/molecules27207009

**Published:** 2022-10-18

**Authors:** Qiang Sun

**Affiliations:** Key Laboratory of Orogenic Belts and Crustal Evolution, Ministry of Education, The School of Earth and Space Sciences, Peking University, Beijing 100871, China; qiangsun@pku.edu.cn

**Keywords:** water, solute, interface, hydrogen bonding, hydrophobic effects

## Abstract

Hydrophobic interactions are involved in and believed to be the fundamental driving force of many chemical and biological phenomena in aqueous environments. This review focuses on our current understanding on hydrophobic effects. As a solute is embedded into water, the interface appears between solute and water, which mainly affects the structure of interfacial water (the topmost water layer at the solute/water interface). From our recent structural studies on water and air-water interface, hydration free energy is derived and utilized to investigate the origin of hydrophobic interactions. It is found that hydration free energy depends on the size of solute. With increasing the solute size, it is reasonably divided into initial and hydrophobic solvation processes, and various dissolved behaviors of the solutes are expected in different solvation processes, such as dispersed and accumulated distributions in solutions. Regarding the origin of hydrophobic effects, it is ascribed to the structural competition between the hydrogen bondings of interfacial and bulk water. This can be applied to understand the characteristics of hydrophobic interactions, such as the dependence of hydrophobic interactions on solute size (or concentrations), the directional natures of hydrophobic interactions, and temperature effects on hydrophobic interactions.

## 1. Introduction

Hydrophobic effects refer to the tendency of nonpolar molecules (or parts of molecules) to be aggregated in water. They are involved in and believed to be the fundamental driving force in many chemical and biological phenomena in aqueous solutions, such as molecular recognition, protein folding, formation and stability of micelles, biological membranes and macromolecular complexes, surfactant aggregation, coagulation, complexation, detergency, and the formation of gas clathrates [1,2,3,4,5]. To date, numerous experimental and theoretical works have been carried out to understand the physical origin of hydrophobic effects.

In general, hydrophobicity is expressed through the empirically calculated logarithm of partition coefficient (logP), which is widely used in drug design and medicinal chemistry [6,7]. Historically, the concept of hydrophobicity arose in the context of the low solubility of non-polar solutes in water. Experimentally, it has been found that the entropy change upon transferring a hydrocarbon from a nonpolar environment into water is large and negative. In 1945, Frank and Evans [8] suggested that the observed loss of entropy was related to the structural changes of liquid water as hydrophobic solutes were dissolved into water. They proposed that a kind of “cage”, consisting of water molecules, was formed around the solute. This ordered water structure, similar to the gas clathrate, was known as the “iceberg” model [8]. Since then, many works [9,10,11,12,13,14,15,16,17] are conducted to measure the water structural changes around hydrophobic surfaces. In accordance with the “iceberg” model, some studies [9,10,11,12] support the existence of increased tetrahedral order around small hydrophobic groups in aqueous solutions. However, the decrease of water structure around hydrophobic groups is also found in many works [13,14,15,16,17]. It is well known that the neutron scattering may be sensitive to the structure of water. From the neutron scattering experimental measurements on aqueous solutions containing tetramethylammonium chloride [13] and methane molecules [15], these do not suggest that water around these hydrophobic solutes may be more ordered than bulk water. To date, there remain strong debates on the “iceberg” structural model.

**Figure 1:**
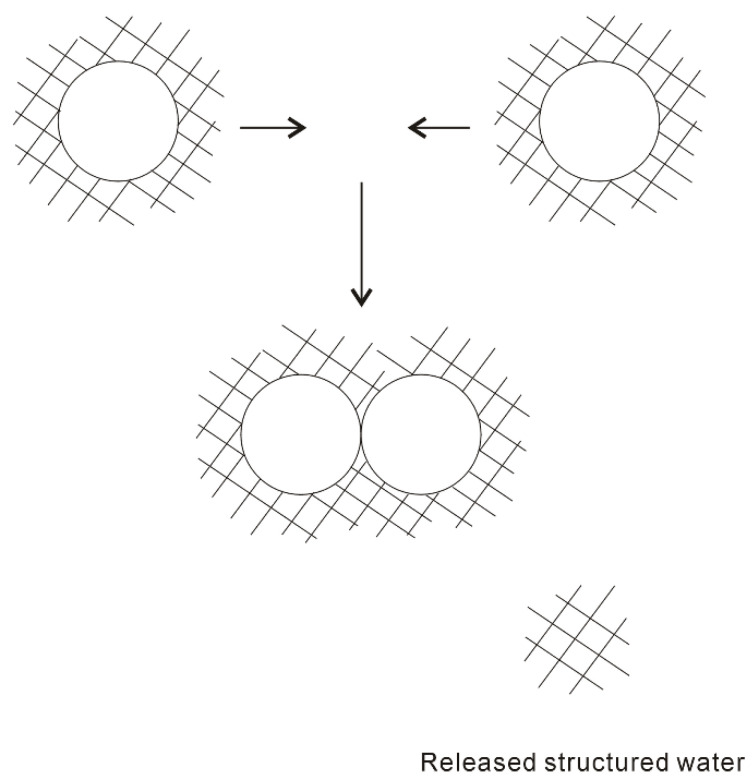
The “iceberg” structural model of hydrophobic effects. The ordered water structure is expected to form around the solute. As two “caged” solutes become together, the “structured” water in the region between them may be returned to the bulk.

In 1959, based on the “iceberg” model, Kauzmann [18] introduced the concept of hydrophobic interactions. When two “caged” hydrophobes come together, the “structured” water between solutes may be released into the bulk (Figure 1), which undoubtedly leads to the increase of entropy. Subsequently, the attractive force between these particles may be related to the entropy increase. Therefore, hydrophobic interactions are classically regarded to be entropy driven. However, the “classic” hydrophobic effects may be in contrast with some works. In Diederich et al. [19] work, they found that complexation of benzene in a cyclophane host molecule was enthalpy driven at room temperature, which was also accompanied with a slightly negative entropy change. Additionally, in Baron, Setny, and McCammon works [20,21], molecular dynamics (MD) simulations are used to investigate the binding in a hydrophobic receptor-ligand system. It is found that the association between the non-polar ligand and binding pocket may be driven by enthalpy and opposed by entropy. The “non-classic” hydrophobic effects are ascribed to the release of weakly hydrogen-bonded water molecules into the more strongly hydrogen-bonded bulk water [22].

In the “iceberg” structural model, water molecules can rearrange around a small hydrophobic solute, without losing their hydrogen bondings. However, as a large solute is embedded into water, hydrogen bondings of water may be broken at the surface of the solute, which may result in an enthalpic penalty. Therefore, hydrophobic interactions depend on the size of dissolved solute. In recent years, a theoretical approach was developed by Lum, Chandler, and Weeks (LCW) [23,24,25,26] to understand the dependence of hydrophobic interactions on solute size. Both the Gaussian density fluctuations related to small size and the physics of interfacial formation related to large size are incorporated in LCW theory [23,24,25,26]. It can be found that the hydration free energy grows linearly with the solvated volume for small solute, but grows linearly with the solvated surface area for large solute [25]. Therefore, the crossover may be expected between small and large regime, which takes place on the nanometer length scale [25].

Liquid water is generally believed to play a vital role in the process of hydrophobic interactions. In cell biology, water may be regarded as an active constituent, rather than a bystander [27,28,29]. According to our recent structural works [30,31,32,33,34,35,36,37,38] on liquid water and air-water interface, hydration free energy is determined. This is utilized to understand the nature of hydrophobic interactions. It is found that hydrophobic interactions may be related to the size of dissolved solute. With increasing the solute size, it is reasonably divided into initial and hydrophobic solvation processes [34]. Additionally, different dissolved behaviors of solutes are expected in initial and hydrophobic solvation processes, such as dispersed and accumulated distributions in aqueous environments. In addition, hydrophobic interactions may be ascribed to the structural competition between interfacial and bulk water [34].

Hydrophobic interactions have long been recognized to be the fundamental driving force in physical chemistry and biochemistry. To understand the origin of hydrophobic interactions, numerous works have been carried out. This review is focused on our current understanding on hydrophobic interactions. Water plays a vital role while solutes are aggregated in solutions. Therefore, it is necessary to study the structure of water, which is included in Section 2. In Section 3, according to the structural works on water and air-water interface, hydration free energy may be determined, and used to investigate the nature of hydrophobic interactions. From this, it is applied to understand the characteristics of hydrophobic interactions, which may be included in Section 4. This work is devoted to studying the dependence of hydrophobic interactions on solute size (or concentrations), the directional natures of hydrophobic interactions, and the temperature effects on hydrophobic interactions.

## 2. Water Structure

Gibbs free energy (ΔG), related to the changes of enthalpy (ΔH) and entropy (ΔS), may be used to study whether a process is likely to take place. Thermodynamically, it is expressed as,

(1) ΔG=ΔH−T⋅ΔS

where ΔH is enthalpy changes, and ΔS is entropy changes. In general, ΔH quantifies the average potential energy between molecules, ΔS measures the order (or intermolecular) correlations of system.

In fact, various interactions between solutes and water may be expected when solutes are embedded into water. The total Gibbs free energy of system is reasonably described as,

(2) $$\Delta G=\Delta G_{\mathrm{Water-water}}+\Delta G_{\mathrm{Solute-water}}+\Delta G_{\mathrm{Solute-solute}}$$

in which ΔG_Water-water_, ΔG_Solute-water_, and ΔG_Solute-solute_, respectively, mean the Gibbs energies are due to water-water, solute-water, and solute-solute interactions. In fact, the solutes are necessarily attracted to approach each other in aqueous solutions before they may be affected by the interactions between them. This is due to the hydrophobic interactions, which accumulate the solutes in the solutions. Therefore, to understand the molecular mechanism of hydrophobic effects, it is important to study the water structure and the effects of solutes on water structure.

Numerous experimental and theoretical works have been carried out to investigate the structure of water. To date, different structural models have been proposed, which are generally partitioned into the mixture and continuum structural models [39,40]. In the mixture model, two distinct types of structures are regarded to simultaneously exist in ambient water. It is likely that the first mixture model was proposed by W.C. Röntgen in 1892, who suggested that liquid water was a mixture of two components, a low-density fluid and a high-density fluid [41]. Since then, various mixture structural models have been proposed [42,43,44]. For the continuum structural model, water is comprised of a random, three-dimensional hydrogen-bonded network, which may be characterized by a broad distribution of O-H···O hydrogen bond distances and angles. However, the water networks cannot be “broken” (or separated into distinct molecular species) as in the mixture model. To date, liquid water is usually regarded as a tetrahedral fluid, which is based on the first coordination number, $N_{c}=4\pi \rho \int_{r_{\min}}^{r_{\max}}\;r^{2}g_{\mathrm{OO}}\left(r\right)\mathrm{dr}$, where ρ means the density of water, r_min_ and r_max_ are the lower and upper limits of integration in oxygen-oxygen radial distribution function, g_OO_(r). For ambient water, Nc is determined to be 4.3 [45] and 4.7 [46], respectively.

Liquid water is usually regarded as an anomalous fluid, which is due to the formation of hydrogen bondings between neighboring water molecules. To understand the physical nature of hydrogen bondings, various theoretical methods have been developed, such as symmetry-adapted perturbation theory (SAPT) [47,48]. From the theoretical calculations [49] on a water dimer ((H_2_O)_2_), besides van der Waals interactions between water molecules, obvious electrostatic interactions can also be found between them. Of course, this is reasonably attributed to the formation of hydrogen bondings between water molecules. Therefore, hydrogen bondings may be ascribed to the electrostatic interactions between the neighboring water molecules.

For an H_2_O molecule, the vibrational normal modes may be 2A_1_+B_1_ [50], which are all Raman active. When hydrogen bonding is formed between neighboring water molecules, this decreases the O···O distance between them, and weakens the O-H covalent bond [51]. The formation of hydrogen bonding may result in OH vibrational frequencies moving towards a low wavenumber (red shift). Therefore, OH vibrations may be sensitive to hydrogen bondings of liquid water, and widely utilized to investigate the structure of water.

**Figure 2:**
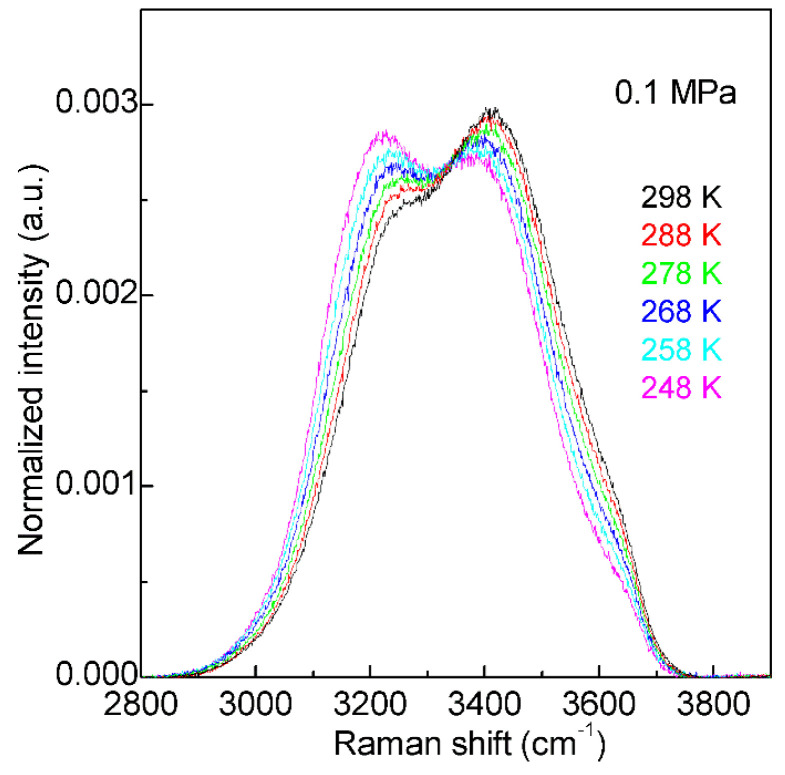
The Raman OH stretching bands of water from 298 to 248 K under 0.1 MPa. Based on normalized intensity, an isosbestic point is found around 3330 cm^−1^.

With decreasing temperature from 298 K to 248 K at 0.1 MPa, based on the normalized intensity, an isosbestic point is found around 3330 cm^−1^ in the Raman OH stretching bands of water (Figure 2). The isosbestic point is the wavelength where a series of spectra cross, and the spectral intensity may keep constant. In mixture structural model, the isosbestic point is generally used to support the two-state behavior of water structure. However, after considering the electric field experienced by the proton projected onto the OH covalent bond, Smith et al. [52] suggested that the isosbestic point was explained through a continuous distribution of local hydrogen-bonded networks, which was due to the increasing distortions around a single-component tetrahedral structural motif. It is noted that, along with the isosbestic point, temperature increase (or adding NaCl) may also lead to the decrease of the second peak at 4.5 Å in g_OO_(r), which undoubtedly means the breakage of tetrahedral hydrogen bondings of water. Therefore, it can be derived that the isosbestic point indicates the structural transition between tetrahedral and non-tetrahedral hydrogen-bonded networks.

**Figure 3:**
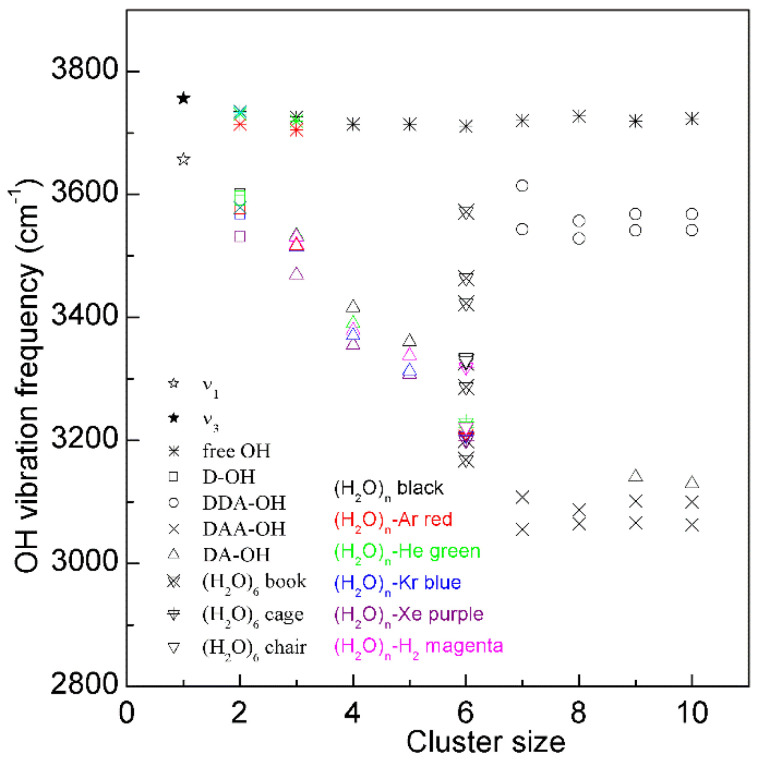
The dependence of the OH stretching frequency on hydrogen bondings of water molecular clusters, (H_2_O)_n_. Different symbols are used to discriminate OH vibrations engaged in various local hydrogen-bonded networks of a water molecule. Various structures of hexamers are also shown.

Many works have been conducted to explain the Raman OH stretching band of water. In fact, water molecular clusters, (H_2_O)_n_, provide an approach to investigate the dependence of OH vibrational frequencies on hydrogen bondings between water molecules. From the theoretical calculations, the stable configurations of water molecular clusters can be determined. In combination with the experimental measurements on OH vibration frequencies of clusters, these may be utilized to unravel the relationship between OH vibrational frequencies and hydrogen-bonded networks. It is found that, when three-dimensional hydrogen-bonded networks occur in water molecular clusters (*n* ≥ 6), different OH vibrational frequencies may correspond to various hydrogen bondings in the first shell of a water molecule (local hydrogen bondings), and the effects of hydrogen bondings beyond the first shell on OH vibrational frequencies may be weak (Figure 3). From this, as three-dimensional hydrogen bondings appear, different OH vibrations may be ascribed to OH vibrations engaged into various local hydrogen bondings [30,32].

**Figure 4:**
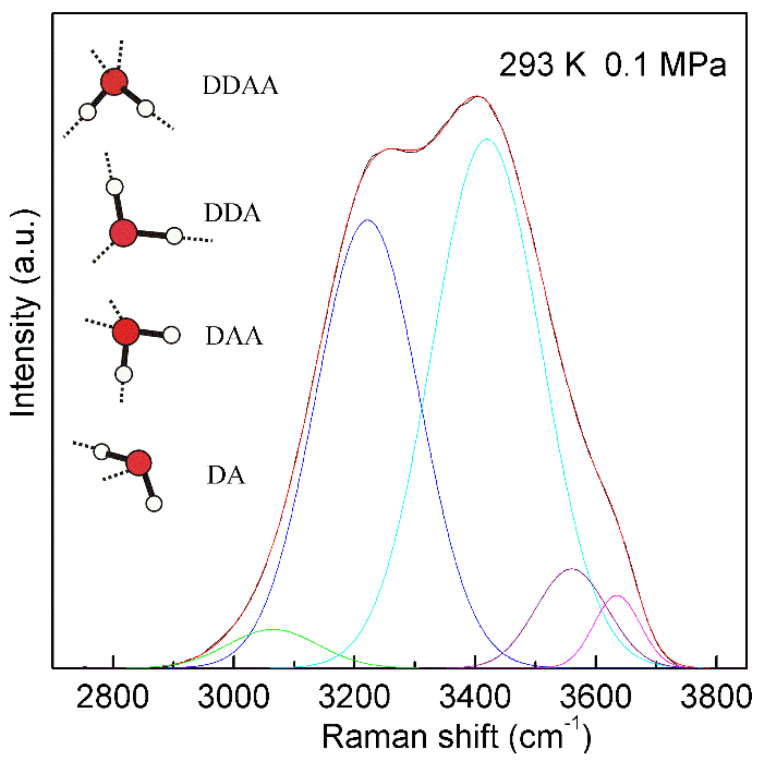
The Raman OH stretching band of ambient water may be deconvoluted into five sub-bands, located at 3041, 3220, 3430, 3572, and 3636 cm^−1^, and assigned to the ν_DAA-OH_, ν_DDAA-OH_, ν_DA-OH_, ν_DDA-OH_, and free OH symmetric stretching vibrations, respectively. At ambient conditions, the main local hydrogen-bonded networks for a water molecule are expected to be DDAA, DDA, DAA, and DA hydrogen bondings. Hydrogen bondings are drawn with dashed lines.

For a water molecule, the local hydrogen-bonded network can be differentiated by whether the molecule forms hydrogen bonds as a proton donor (D), proton acceptor (A), or a combination of both with neighboring molecules. Under ambient conditions, the main local hydrogen bonding motifs for a water molecule can be classified as DDAA (double donor-double acceptor), DDA (double donor-single acceptor), DAA (single donor-double acceptor), and DA (single donor-single acceptor) (Figure 4). For ambient water, the Raman OH stretching band may be fitted into five sub-bands, which can be assigned to the ν_DAA-OH_, ν_DDAA-OH_, ν_DA-OH_, ν_DDA-OH_, and free OH symmetric stretching vibrations, respectively (Figure 4). Therefore, at ambient conditions, different local hydrogen bondings may be expected around a water molecule.

From the Raman spectroscopic studies [30,31,32] on ambient water, the local statistical model (LSM) is proposed. This suggests that a water molecule interacts with the neighboring water molecules (in the first shell) through different local hydrogen bondings. Of course, it is different from continuum structural models of ambient water. Additionally, according to the mixture structural model, water has been considered as a mixture of two distinct types of structures. Therefore, different spatial distributions may be necessary for various structural types, and sharp boundary is expected between them. In mixture model, water structure may be heterogeneous. Additionally, according to recent X-ray experimental studies [53,54], these mean that liquid water is heterogeneous at ambient conditions, and this becomes enhanced in the supercooled region. However, based on LSM of water structure, various local hydrogen bondings are expected for a water molecule at ambient conditions. This indicates that it is impossible to find a sharp phase boundary between various structural motifs, and the structure of ambient water may be homogeneous. Of course, it is different from the mixture structural model. In addition, based on LSM model, the local hydrogen-bonded networks of a water molecule may be affected by pressure, temperature, dissolved salt, and a confined environment.

**Figure 5:**
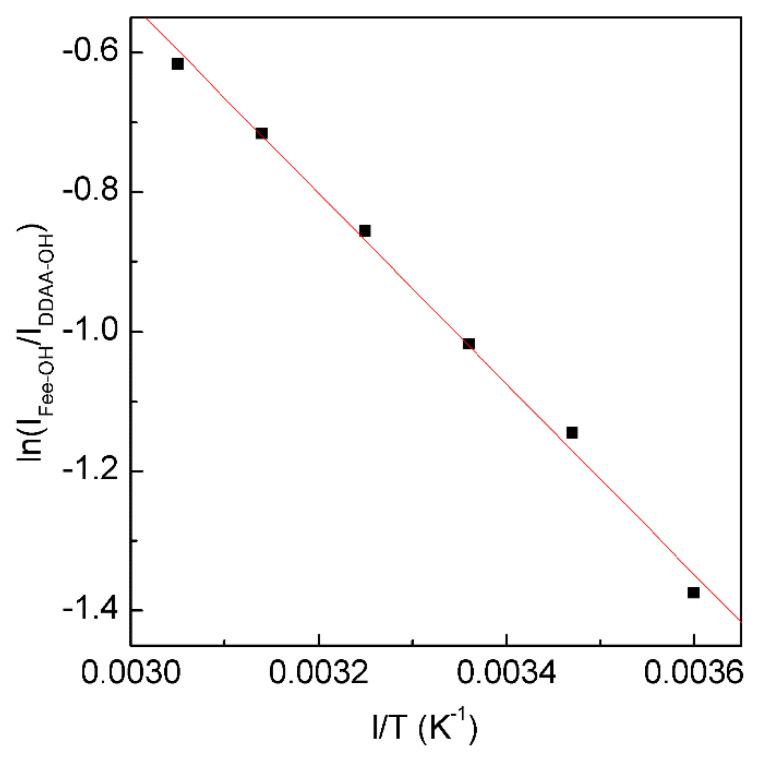
The dependence of ln(I_Free-OH_/I_DDAA-OH_) on 1/T. The solid line represents linear fit (R^2^ = 0.9975) with a slope of −∆H/R. This is used to determine the thermodynamic characteristics of tetrahedral hydrogen bonding.

According to the explanation on Raman OH stretching band of water [30,31,32], ν_DDAA-OH_ is due to OH vibration engaged in DDAA (tetrahedral or “unbroken”) hydrogen bonding, and ν_Free-OH_ is free OH symmetric stretching vibration. From the van’t Hoff equation, this may be applied to calculate the thermodynamic functions of tetrahedral (DDAA) hydrogen bonding (Figure 5),

(3) $$\ln\left(\frac{I_{\mathrm{Free-OH}}}{I_{\mathrm{DDAA-OH}}}\right)=-\frac{\Delta H}{\mathrm{RT}}+\frac{\Delta S}{R}$$

From the Raman OH stretching bands from 298 K to 248 K, the enthalpy (∆H) and entropy (∆S) from tetrahedral hydrogen bonding to free water are calculated to be 11.35 kJ/mol and 29.66 J/mol, respectively.

Additionally, based on the structural explanation on the Raman OH stretching band of water, it is derived that the isosbestic point (Figure 2) may indicate the structural equilibrium between different local hydrogen bondings around a water molecule. This is expressed as follows,

(4) DAA+DDAA=DA+DDA+Free OH

With decreasing temperature from 298 to 248 K at 0.1 MPa, this decreases the intensity of the high wavenumber sub-bands (>3330 cm^−1^), but increases the intensity of the low wavenumber sub-bands (<3330 cm^−1^) (Figure 2). Therefore, temperature decrease may enhance the probability to form tetrahedral hydrogen-bonded networks around a water molecule, which is related to the nucleation of ice. In fact, according to recent MD simulations [55,56,57] on homogeneous ice nucleation, the ordered ice-like intermediate can be found in the supercooled water, and ice nucleation is found to occur in the low-mobility regions [56,57]. Additionally, this intermediate phase has also been found in other theoretical simulations [58,59,60] during the nucleation from supercooled liquids, such as Lenard-Jones hard spheres and metals. From these, a non-classical pathway, rather than classical nucleation theory (CNT), is proposed in order to understand the nucleation mechanism in supercooled liquids.

Water has many unusual thermodynamic and dynamic properties, both in pure form and as a solvent. Additionally, these anomalous behaviors may be strongly enhanced in the supercooled state, such as thermal expansion, isothermal compressibility, etc. To explain the origin of the anomalous behaviors of supercooled water, many theories are proposed, such as the stability limit (SL) conjecture [61], the liquid-liquid critical-point (LLCP) hypothesis [62], the singularity-free (SF) model [63], and the critical-point free scenario [64]. Recently, numerous works [54,65,66,67,68,69] have been carried out to demonstrate the existence of second critical point in LLCP. In fact, LLCP is based on the mixture model of water structure. In LLCP, two liquid phases are expected in water, such as low-density water (LDW) and high-density water (HDW), which may interconvert through a first-order liquid-liquid transition terminating at the second critical point in the supercooled regime. In this hypothesis, the anomalous behavior of water is due to the fluctuations emanating from the LLCP.

With decreasing temperature from 298 to 248 K at 0.1 MPa (Figure 2), this increases the probability to form the tetrahedral (DDAA) hydrogen bondings in supercooled water. In comparison with bulk water, DDAA hydrogen bondings have a larger volume, and lower entropy [32]. This indicates that the formation of tetrahedral hydrogen bondings in a supercooled regime may be used to explain the anomalies of supercooled water, such as the thermal expansion. From statistical mechanics, the thermal expansion may be related to the correlation of the fluctuations of volume and entropy, $\alpha_{P}=\frac{1}{V}{\left(\frac{\partial V}{\partial T}\right)}_{P}=\frac{1}{\mathrm{TV}}\langle \Delta S\Delta V\rangle$. For most fluids, with increasing volume, it is also accompanied with an increase of entropy. However, regarding water below TM (temperature of maximum density), the fluctuations of volume and entropy may be anti-correlated. From the above, it is more reasonable to explain the anomalous properties of supercooled water from the SF model [63]. Further study is necessary.

**Figure 6:**
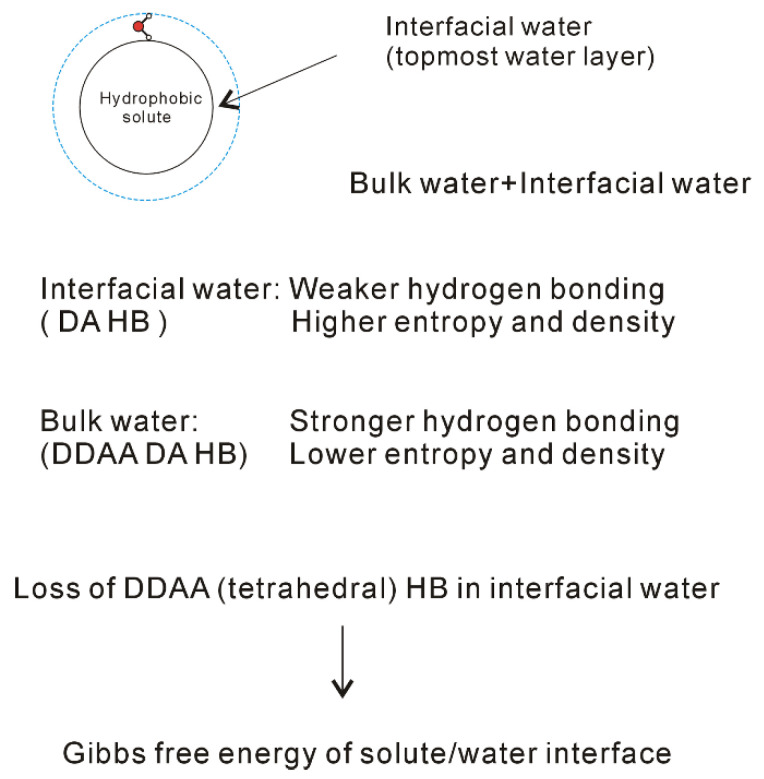
Structural changes across the solute-water interface. The dissolved solute mainly affects the structure of interfacial water (topmost water layer at the interface).

When a solute is dissolved into liquid water, an interface appears between the solute and water. Therefore, the dissolved solute undoubtedly affects the structure of water. At ambient conditions, the OH vibration is mainly related to the local hydrogen-bonded networks of a water molecule. It is derived that the dissolved solute may mainly affect the structure of topmost water layer at the solute-water interface (interfacial water) (Figure 6). In fact, this is in accordance with other studies [70,71,72,73,74] on the structure and dynamics of water around ions. These mean that the effects of dissolved ions on water structure may be largely limited to the first solvation shell. Therefore, as a solute is embedded into water, it may be divided into interfacial and bulk water (Figure 6). Regarding the effects of solutes on water structure, these may be related to the solute-water interfaces. In other words, it is related to the water in confined environments. In our Raman spectroscopic study [75] on confined water, it can be found that, in comparison with DDAA (tetrahedral) hydrogen bondings, DA hydrogen bondings are expected to form under confined environments. Therefore, it can be derived that the DA hydrogen bonding may tend to form at the solute-water interface.

In theory, vibrational sum frequency generation (SFG) spectroscopy provides a unique and powerful method to investigate the surface and interface at the molecular level. Many SFG experimental works have been conducted to study the structure of air-water interface [33,76,77,78,79]. In recent years, phase sensitive sum-frequency generation (PS-SFG) spectroscopy has been developed by Shen et al. [80,81]. From the PS-SFG measurements, Imχ(2) may be directly determined, and the sign of the imaginary part of χ(2) may be utilized to reflect the water molecular dipole direction. Based on our SFG study on the air-water interface [33], no tetrahedral (DDAA) hydrogen bonding is expected to form in the interfacial water, and an obvious structural difference may be found across the air-water interface.

From the Raman spectroscopic studies [30,31,32], DDAA (tetrahedral) and DA are the predominant hydrogen-bonded networks in ambient water. Additionally, they are related to the structural changes across the solute-water interface (Figure 6). It is necessary to understand the characteristics of DDAA and DA hydrogen bondings. For ambient water, the DDAA-OH, located around 3220 cm^−1^, lies at a lower frequency than DA-OH (3430 cm^−1^) (Figure 4). This indicates that, in comparison with DA hydrogen bonding, the formation of DDAA structural motif may result in higher hydrogen bonding energies. Additionally, it is necessary for the interacting molecules to lie in specific relative orientations to form hydrogen bondings between neighboring water molecules, therefore DDAA (tetrahedral) is expected to own the lower entropy rather than DA hydrogen bonding. In addition, based on the experimental measurements [82] and theoretical simulations [83,84], higher water density can be found at the solute-water interface, which may be related to the formation of DA in interfacial water. Therefore, higher density is expected for DA as a structural motif than it is for DDAA hydrogen bonding. In comparison with DDAA (tetrahedral) hydrogen-bonded network, the DA structural motif owns a lower enthalpy, and a higher entropy and density.

The dissolved solute mainly affects the structure of interfacial water. Additionally, DA structural motif tends to form at the interface between solute and water. In other words, this means that the loss of tetrahedral hydrogen bonding may be related to the formation of solute-water interface (Figure 6). Certainly, this is different from the “iceberg” structural model proposed by Frank and Evans [8]. In history, the “iceberg” structural model was proposed in order to understand the large and negative entropy while the simple solutes were dissolved in water. In Frank and Evans’ work [8], an increase in the structure of water was utilized to explain the negative entropy incurred from the dissolved non-polar molecules. However, they did not explain why the hydrophobic solute could lead to increase of the order of the system. In fact, this may be due to the transition from interfacial to bulk water as the solutes are dissolved in solutions, which leads to the increase of DDAA hydrogen bondings in water.

The effects of dissolved solute on water structure are mainly limited within the interfacial water layer (Figure 6). Additionally, the formation of solute-water interface is due to the loss of tetrahedral hydrogen bonding in interfacial water. Therefore, as the ratio of interfacial water layer to volume is obtained, this may be utilized to calculate the Gibbs free energy of the interface between solute and water. From this, it is reasonably expressed as,

(5) $$\Delta G_{\mathrm{Solute-water}}=\Delta G_{\mathrm{DDAA}}\cdot R_{\mathrm{Interfacial water/volume}}\cdot n_{\mathrm{HB}}$$

in which ∆G_DDAA_ means the Gibbs free energy of tetrahedral hydrogen bonding, R_Interfacial water/volume_ is the molecular number ratio of interfacial water layer to volume, and n_HB_ means the average hydrogen bonding number per molecule. For tetrahedral hydrogen bonding, n_HB_ is equal to 2.

## 3. Hydrophobic Effects

**Figure 7:**
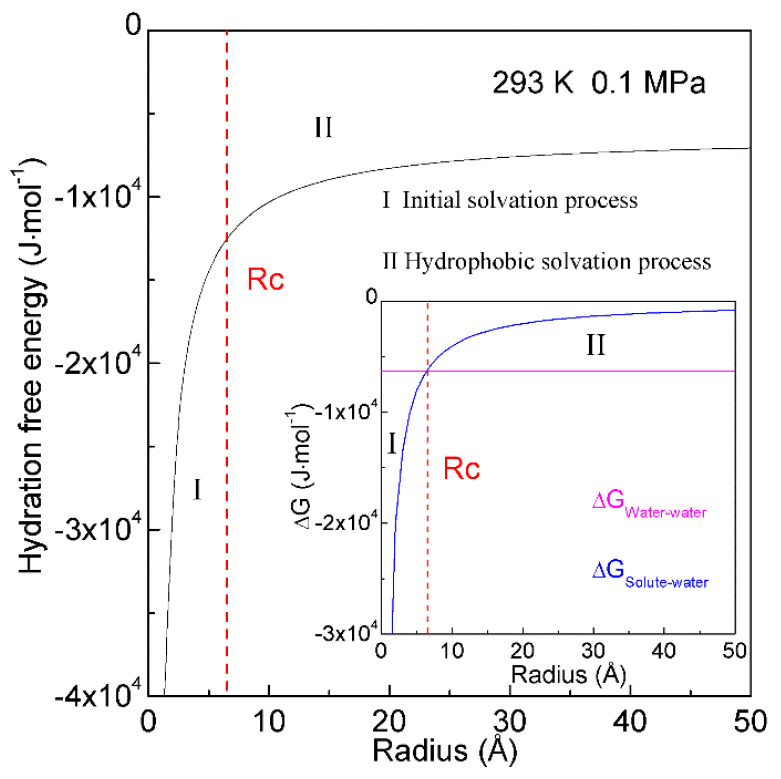
Hydration free energy at 293 K and 0.1 MPa. Hydration free energy is related to the size of solute, and critical radius (Rc) is expected. With increasing the solute size, it is divided into initial and hydrophobic solvation processes.

When a solute is embedded in liquid water, it is thermodynamically equivalent to form the solute-water interface. After the solute is treated as a sphere, the R_Interfacial water/volume_ is 4·r_H2O_/R, in which R means the radius of solute. The hydration free energy is the free energy associated with the transfer of solute from vacuum to water. Therefore, as the sphere solute is dissolved into water, hydration free energy may be described as (Figure 7),

(6) $${\Delta G}_{\mathrm{Hydration}}={\Delta G}_{\mathrm{Water-water}}+{\Delta G}_{\mathrm{Solute-water}}={\Delta G}_{\mathrm{Water-water}}+\frac{8\cdot {\Delta G}_{\mathrm{DDAA}}\cdot r_{H2O}}{R}$$

in which ΔG_Water-water_ is the Gibbs free energy of water, r_H2O_ is the average radius of a H_2_O molecule. For water at 293 K and 0.1 MPa, Gibbs free energy (ΔG_Water-water_) is −1500 cal/mol [85]. Additionally, the average volume of a water molecule is 3 × 10^−29^ m^3^ at ambient conditions. After the water molecule is regarded as a sphere, the corresponding diameter is 3.8 Å, and r_H2O_ is 1.9 Å. In addition, the Gibbs free energy of tetrahedral hydrogen bonding (ΔG_DDAA_) is calculated to be −2.66 kJ/mol at 293 K and 0.1 MPa.

In thermodynamics, the lower the hydration free energy, the more stable the system is. Because hydration free energy is the sum of ∆G_Water-water_ and ∆G_Solute-water_, it may be dominated by the Gibbs energy of bulk water (∆G_Water-water_) or interfacial water (∆G_Solute-water_). Of course, it is related to the size of dissolved solute. Therefore, the structural transition may be expected to occur as ∆G_Water-water_ being equal to ∆G_Solute-water_,

(7) $$\Delta G_{\mathrm{Water-water}}=\Delta G_{\mathrm{Solute-water}}\;(\mathrm{Rc}=\frac{8\cdot \Delta G_{\mathrm{DDAA}}\cdot r_{H_{2}O}}{\Delta G_{\mathrm{Water-water}}})$$

in which Rc means the critical radius of dissolved solute [34]. In our recent study [34], Rc is determined to be 6.5 Å at 293 K and 0.1 MPa (Figure 7).

**Figure 8:**
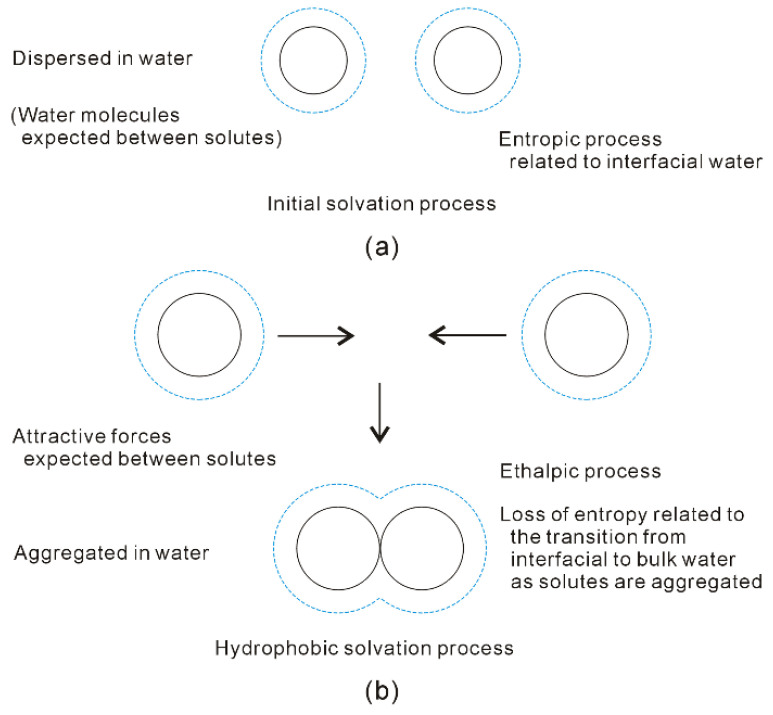
Different dissolved behaviors of solutes in aqueous solutions may be expected in initial (**a**) and hydrophobic (**b**) solvation processes.

With increasing the solute size, it is reasonably divided into the initial (ΔG_Water-water_ < ΔG_Solute-water_) and hydrophobic (ΔG_Water-water_ > ΔG_Solute-water_) solvation processes. The Gibbs free energy between solute and water (ΔG_Solute-water_) is inversely proportional to the solute size (1/R), which is related to the ratio of surface area to volume. Therefore, various dissolved behaviors of solutes in aqueous solutions may be expected in initial and hydrophobic processes, which may be related to the solute size (or concentrations) (Figure 8).

In the initial solvation process, ΔG_Solute-water_ is lower than ΔG_Water-water_ (both of them are negative), or the solute size is smaller than Rc. Therefore, hydration free energy is dominated by the Gibbs free energy of interfacial water (ΔG_Solute-water_) [34,36]. To become more thermodynamically stable, this is fulfilled through maximizing the |ΔG_Solute-water_|. In other words, it is achieved through maximizing the ratio of surface area to volume of dissolved solutes. Therefore, the solutes may be dispersed in aqueous solutions, and water molecules are found between them (Figure 8). In addition, hydration free energy is proportional to the volume (or concentrations) of dissolved solutes.

Additionally, the dissolved solute mainly affects the hydrogen-bonded networks of interfacial water, DA hydrogen bondings tend to form within interfacial water layer [34]. In the initial solvation process, hydration free energy may be related to DA hydrogen bondings. In comparison with DDAA (tetrahedral) structural motif, DA hydrogen bonding owns weaker hydrogen bonding energy, and higher entropy. Therefore, the driving force may be thermodynamically ascribed to the increase of entropy arising from interfacial water [34].

In hydrophobic solvation process, Gibbs free energy of interfacial water is higher than bulk water (ΔG_Solute-water_ > ΔG_Water-water_). To be more thermodynamically stable, this may be fulfilled through maximizing |ΔG_Water-water_|. Indeed, this is accompanied with the minimization of Gibbs free energy of interfacial water (|ΔG_Solute-water_|). From the above, the ΔG_Solute-water_ is related to the ratio of surface area to volume of solutes. It can be derived that the dissolved solutes may be aggregated in solutions in order to maximize the hydrogen bondings of water [34,36]. Therefore, the “attractive” forces may be expected between solutes in hydrophobic solvation process (Figure 8).

Due to the existence of DDAA (tetrahedral) hydrogen bondings in bulk water, this leads to ΔG_Water-water_ being lower than ΔG_Solute-water_. In comparison with DA hydrogen bonding, tetrahedral hydrogen-bonded networks own the stronger hydrogen bonding energy, and lower entropy [32]. Regarding the “attractive” force between solutes, it may be ascribed to be an enthalpic process, which is related to DDAA (tetrahedral) hydrogen bondings in bulk water. As the solutes are aggregated in water, they are also accompanied with the loss of entropy, which is related to the transition from interfacial to bulk water (Figure 8). Additionally, hydration free energy is proportional to the surface area of dissolved solutes.

**Figure 9:**
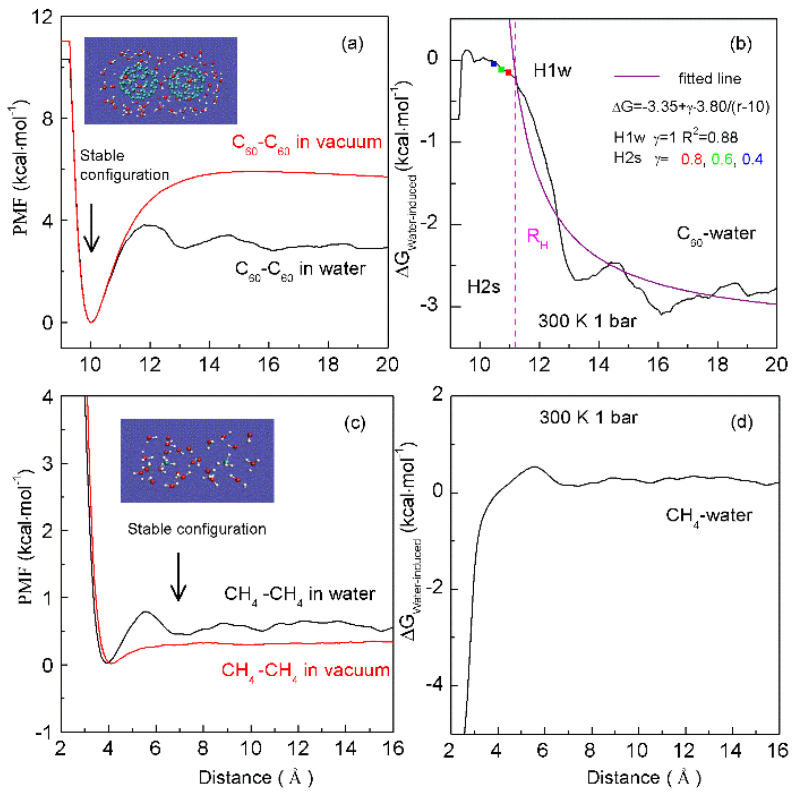
The dependence of hydrophobic interactions on solute size. Based on the calculated potential of mean forces (PMFs) between C_60_ fullerenes in water and under vacuum (**a**), the water-induced PMF between C_60_ fullerenes is determined (**b**). It is fitted as, ΔG = −3.35 + γ·3.80/(r − 10). During the H1w process, γ = 1. In the H2s process, solutes become contact in solutions. The fitted results at various γ (0.8, 0.6, 0.4) are drawn in squares. From the PMFs between CH_4_ dimers in water and under vacuum (**c**), these are used to determine the water-induced PMF between CH_4_ dimers (**d**). Stable configurations are also shown.

The solutes may be “dispersed” or “accumulated” in water, which is related to the size of the solute. As two same sphere solutes are embedded into water, hydration free energy may be related to the separation between them. Therefore, the corresponding Rc is 3.25 Å at 293 K and 0.1 MPa [36]. At ambient conditions, hydrophobic interactions may be expected as the solute radius is larger than 3.25 Å (>3.25 Å). This is demonstrated by our recent MD simulations [36] on two C_60_ fullerenes in water, and a pair of CH_4_ molecules in water (Figure 9). Based on the calculated potential mean forces (PMFs), the water-induced contributions may be determined for C_60_-C_60_ in water, and CH_4_-CH_4_ in water, respectively. It seems that there exists the “attractive” force between two fullerenes (Figure 9). This is due to the radius of C_60_ fullerene is larger than Rc. Because the CH_4_ radius is less than 3.25 Å, water molecules are expected to exist in the region between two CH_4_ molecules. The dissolved CH_4_ molecules tend to be engaged in the solvent-separated conformation (Figure 9). In other words, as the CH_4_ molecules are pushed together, this leads to the water molecules between them to be expelled into bulk water, which may be less thermodynamically stable. Therefore, as the CH_4_ molecules are associated in water, the “repulsive” forces between them may be expected. This is in agreement with the Ashbaugh et al. MD simulations [86].

**Figure 10:**
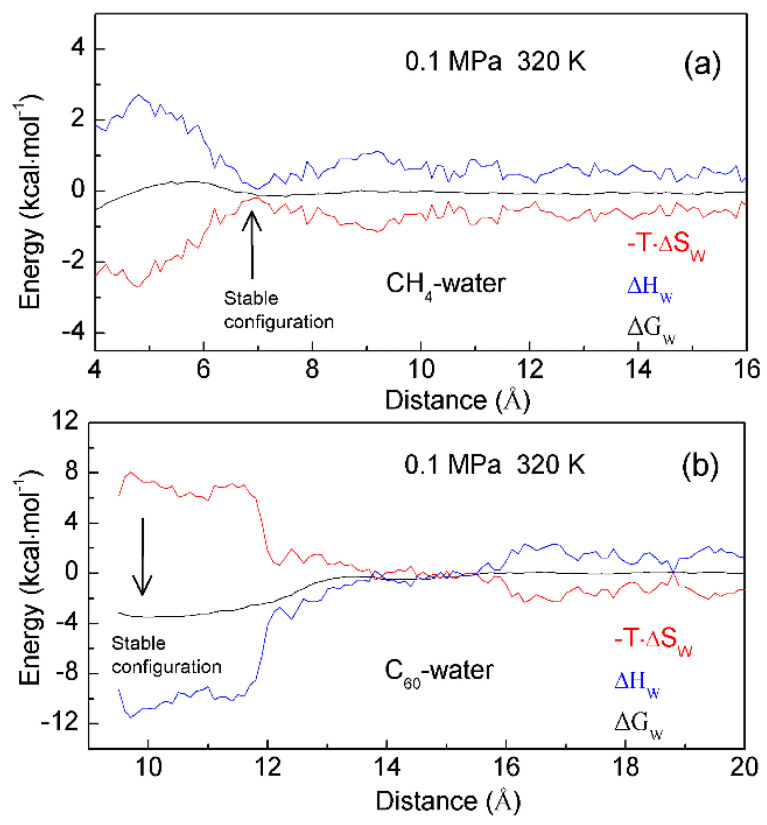
The thermodynamic characteristics of hydrophobic interactions. Based on the calculated PMFs of C_60_-C_60_ fullerenes and CH_4_-CH_4_ in water at different temperatures (300 K, 320 K and 340 K), water contributions to Gibbs energy (ΔG_W_) are determined, which are used to calculate the enthalpic (ΔH_W_) and entropic (-T·ΔS_W_) contributions. Different thermodynamic characteristics may be expected in initial (**a**) and hydrophobic (**b**) solvation processes.

To investigate the thermodynamic properties of hydrophobic interactions, the PMFs can be determined through MD simulations on C_60_-C_60_ fullerenes in water, and CH_4_-CH_4_ in water at different temperature (300 K, 320 K and 340 K) (Unpublished data). Based on the calculated ΔG_Water-induced_ for C_60-_C_60_ in water, and CH_4_-CH_4_ in water, these can be applied to determine the thermodynamic functions as the solutes are associated in water (Figure 10). To be more thermodynamically stable, the two CH_4_ molecules are engaged in the solvent-separated conformation. In thermodynamics, it is driven by the entropy contributions related to interfacial water (Figure 10). However, the two fullerenes tend to be accumulated in solutions. Thermodynamically, it is dominated by enthalpy related to maximizing the hydrogen bondings of water. Additionally, this is also accompanied with the loss of entropy. Indeed, this is related to the transition from interfacial to bulk water as the fullerenes are aggregated in solutions, especially as the distance between them is less than 13 Å (Figure 10). Therefore, various thermodynamic driving forces may be expected in initial and hydrophobic solvation processes, which may be also in agreement with the above structural studies.

In addition, enthalpy-entropy compensation (EEC) has been attracting attention because it is involved in many research fields [87,88,89,90,91], especially for the understanding of molecular recognition and drug design. In thermodynamics, EEC means that, if, for the particular reaction, ΔH and ΔS are changing in one direction (either increasing or decreasing), their changes that are transformed into ΔG are mutually compensated, and there is little change in the value of ΔG. To understand the nature of EEC, many works have been carried out. This means that EEC is real, very common, and a consequence of the properties of liquid water. Based on the calculated thermodynamics functions (Figure 10), hydrophobic interactions are closely related to EEC. Therefore, water plays a vital role in the process of EEC. Of course, this has been demonstrated in the work of Gilli et al. [91].

The dissolved solutes mainly affect the structure of interfacial water. Therefore, the effects of solutes on water structure may be related to the surfaces of solutes to be available for interfacial water. Owing to hydrophobic interactions, the dissolved solutes are attracted and tend to be aggregated in aqueous solutions in order to maximize the hydrogen bondings of water. In fact, the solutes coming into contact undoubtedly leads to the decrease of the solute surfaces available for interfacial water. Therefore, the Gibbs free energy of interfacial water may be described as,

(8) $$\Delta G_{\mathrm{Interfacial water}}=\gamma \cdot \Delta G_{\mathrm{Solute-water}}$$

where γ is named as the geometric factor [36]. It is proposed to reflect the changes of solute surfaces while solutes are accumulated in water. Naturally, the solutes are rarely rigid. As they are dissolved in solutions, this may be accompanied by the changes of solute volume. From this, γ may be generally expressed as,

(9) γ=(Surface areaVolume)Aggregate(Surface areaVolume)Non-aggregate=f(1rSeparation)

in which r_Separation_ means the distance between solutes. When the solutes come into contact, the corresponding distance between them is termed ‘the hydrophobic radius’ (R_H_) [36]. As the solutes are aggregated in water, it may be divided into H1w and H2s hydrophobic solvation processes, respectively (Figure 9).

Water molecules may be found between the dissolved solutes in H1w hydrophobic process, the separation between solutes is larger than R_H_ (>R_H_), or γ is 1 [36]. Due to hydrophobic interactions, the solutes are attracted to approaching each other. This decreases the distance between the solutes, and the water molecules in the region between them are expelled into bulk water. Therefore, hydrophobic interactions are fulfilled by the rearrangement of water molecules. Additionally, energy barriers may be expected in the H1w process, due to the expelled water molecules. Thermodynamically, the dissolved solutes are expected to approach each other in the direction with the lowest energy barrier, in which less water molecules may be expelled. From the above, the directional nature may be expected in the H1w hydrophobic process [37].

As the dissolved solutes come into contact in the H2s hydrophobic process (<R_H_), γ is less than 1 (γ < 1). The aggregation of solute surface undoubtedly leads to the decrease of solute surface to be available for interfacial water. ΔG_Solute-water_ is proportional to the ratio of surface area to volume of the solutes. To become more thermodynamically stable, the solutes may be accumulated to minimize their ratio of surface area to volume. In fact, this may be demonstrated by MD simulations on graphite sheets [92,93] and carbon nanotubes (CNTs) [93,94] in solutions. Therefore, in the H2s solvation process, the directional nature of hydrophobic interactions is also expected. From the above, different directional natures are found in H1w and H2s processes, which may be discussed in the following section.

**Figure 11:**
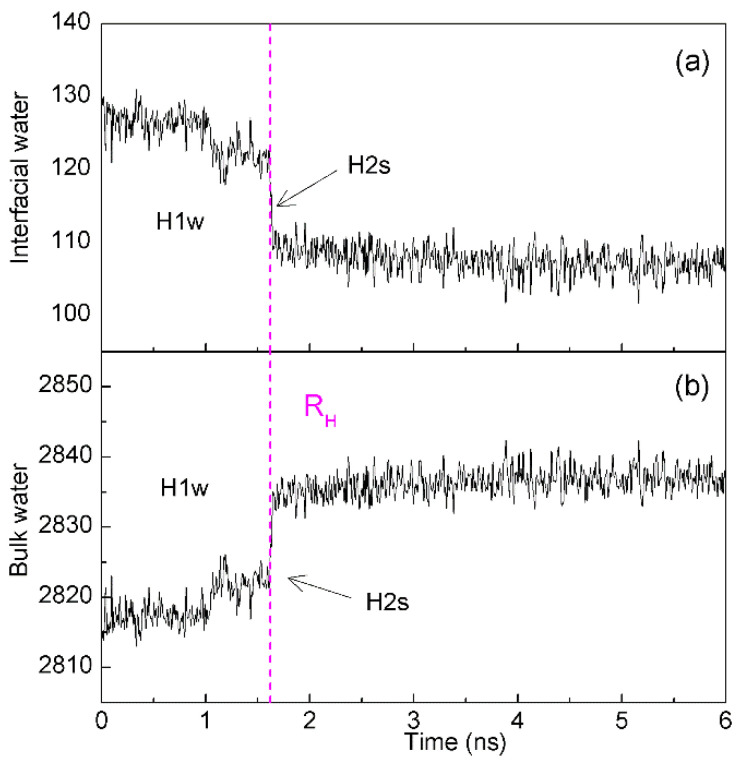
The changes of interfacial (**a**) and bulk (**b**) water during two C_60_ fullerenes are aggregated in solutions. The dashed line represents the corresponding time of R_H_.

While the solutes are accumulated in solutions, the dewetting transition process, which is similar to the liquid-gas phase transition, may be observed (Figure 11). In 1995, according to MD simulations, Wallqvist and Berne [95,96] found the depletion of water between two nanoscale hydrophobic particles. Since then, water dewetting has been observed in many MD simulations on nanotubes and plates in water [97,98], water-protein interfaces [99], and collapsing polymers [100]. Generally, the dewetting is believed to lead to the long-range hydrophobic attraction between solutes. Based on our recent study [36], dewetting may be closely related to H2s hydrophobic process, in which a single water layer between solutes may be expelled, and solutes become contact in solutions. In addition, dewetting is also related to the intermolecular interactions between solutes.

Hydrophobic effects are usually defined as the tendency of non-polar solutes to be aggregated in aqueous environments. From our recent studies [34,36,37] on hydrophobic effects, this may be reasonably described as the tendency for minimizing the ratio of the surface area to the volume of the solutes in order to maximize the hydrogen bondings of water. In fact, this is due to the dissolved solute mainly affecting the structure of interfacial water, as the hydrogen bondings of interfacial water are weaker than bulk water. Additionally, it is found that hydrophobic interactions are reasonably attributed to the structural competition between interfacial and bulk water. Therefore, it is reasonable to regard the hydrophobicity as “effects” rather than “bonds” [34,36,37].

## 4. Characteristics of Hydrophobic Interactions

Owing to hydrophobic interactions, the solutes are attracted to and tend to be aggregated in aqueous solutions to maximize the hydrogen bondings of water. The dissolved solutes mainly affect the structure of interfacial water. Therefore, hydrophobic interactions may be closely related to the geometric characteristics of solute, such as the solute size (or concentrations), the shape of solute, and the relative orientation between dissolved solutes. Additionally, hydrophobic interactions are due to the structural competition between interfacial and bulk water. In fact, water structure may be affected by many factors, such as temperature, pressure, dissolved salt, confined environments, etc. Therefore, these factors may also affect the hydrophobic interactions. In this review, it is focused on the dependence of hydrophobic interactions on solute size (or concentrations), the directional natures of hydrophobic interactions, and the temperature effects on hydrophobic interactions.

### 4.1. Dependence of Hydrophobic Interactions on Solute Size (or Concentrations)

With increasing the solute size, it may be divided into initial and hydrophobic solvation processes. Additionally, different dissolved behaviors of solutes may be expected in initial and hydrophobic solvation processes, such as the “dispersed” or “accumulated” distributions in solutions, which may be related to the solute size (or concentrations). In hydrophobic solvation process, the “attractive” forces are expected between the dissolved solutes. To maximize the hydrogen bondings of water, the dissolved solutes tend to be accumulated to form the aggregate in aqueous environments. Regarding the strength of hydrophobic interactions, this may be related to the size of aggregate. In our recent study [38], this is utilized to understand the origin of intermediate phase, which may be found before solute nucleation occurs in aqueous solutions.

In general, classical nucleation theory (CNT) is employed to understand the mechanism of nucleation. In the nucleation process, it is assumed that the free energy may be divided into a favorable term, and an unfavorable term. They are respectively related to the number of particles in the nucleus, and the dividing surface between the nucleus and the solution. In CNT, the free energy difference may be expressed as,

(10) $$\Delta G_{\mathrm{CNT}}=-\Delta \mu \cdot n+\gamma \cdot s$$

in which n is the molecular number of the crystal phase, Δμ represents the difference of chemical potential between crystal and liquid phase, γ means the surface tension, and s is the surface of the nucleus. From this, the critical nucleus (Nc) is expected in the process of nucleation.

The nucleation of crystals from solution is a ubiquitous process, which plays an important role in many research fields. To unravel the nucleation mechanism, many experimental approaches [101,102,103,104,105] have been developed and applied to study the nucleation mechanism of crystal in aqueous solutions. Different from CNT, the intermediate phase is found before the nucleation takes place in solutions, such as amorphous calcium carbonate (ACC) [104,105]. Therefore, the intermediate phase is regarded to be related to the solidification pathway of dissolved solutes. To understand the non-CNT pathway, various models are put forth, such as the two-step nucleation model [106,107]. According to the two-step nucleation mechanism, amorphous nuclei (intermediate phase) may be formed in the first step. Of course, it is necessary to overcome the energy barrier to form intermediate phases in solutions. In the second step, the nucleation of crystal is expected to take place in the middle of the amorphous phase. In comparison with direct nucleation from solution, the lower energy barrier is needed to overcome in the second step.

Regarding the non-CNT pathway, it is important to understand the origin of intermediate phase. Many theoretical simulations [38,108,109,110,111,112,113,114,115] have been carried out to study the mechanism of NaCl nucleation from the solutions. In a Giberti et al. study [108], a wurtzite-like polymorph was found in the nucleation process, which was regarded as an intermediate route during NaCl nucleation in the solution. Based on the Chakraborty and Patey MD simulation [113], an unstructured dense NaCl nucleus was found and transformed into the rock salt structure. These seem to support the two-step nucleation mechanism. According to our recent MD simulations [38], it is found that the dissolved behaviors of NaCl in water may be related to ion concentrations. With increasing the concentrations, the dissolved Na^+^ and Cl^−^ ions may be associated to form the aggregate in solutions. However, no energy barrier is necessary to overcome this during the formation of the aggregate, which is different from the two-step nucleation mechanism. Additionally, as the ion aggregate is formed in solution, this is accompanied with the maximization of hydrogen bondings of water. In combination with our recent works [34,36], this is reasonably ascribed to the hydrophobic interactions. Compared with CNT, the formation of solute aggregate may lower the barrier height of nucleation and affect the nucleation mechanism of NaCl crystal in water.

**Figure 12:**
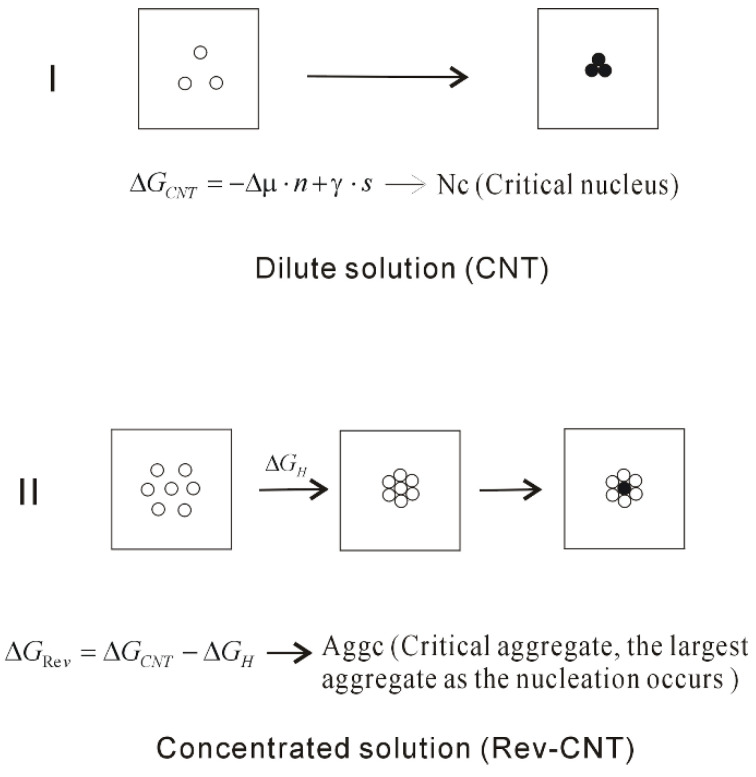
The homogeneous nucleation mechanism of dissolved solutes in water. The dissolved behaviors of solutes in water are related to the ion concentrations, which affect the nucleation mechanism of crystal in water. Due to the formation of solute aggregate, this lowers the height of nucleation barrier.

With increasing ion concentrations, the dissolved ions tend to be aggregated to form the intermediate phase in solutions, which may be related to hydrophobic interactions. However, this is not considered in CNT theory. To explain the nucleation mechanism in solutions, it may be necessary to take into account the hydrophobic interactions related to the formation of aggregate. From this, CNT is reasonably revised as (Rev-CNT) (Figure 12),

(11) $$\Delta G_{\mathrm{Rev}}=\Delta G_{\mathrm{CNT}}-\Delta G_{H}$$

where ΔG_H_ represents the hydrophobic interactions related to the formation of intermediate phase. Additionally, different from the Nc in CNT, the critical aggregate (Agg_C_) may be expected, which is the largest aggregate as the nucleation takes place in the middle of intermediate phase.

Generally, nucleation processes can be classified as homogeneous and heterogeneous [116]. For heterogeneous nucleation, it is formulated within the CNT framework, which is expressed as,

(12) $$\Delta G_{\mathrm{Heterogeneous}}=\Delta G_{\mathrm{Homogeneous}}\cdot f\left(\theta \right)$$

in which f(θ) is the shape factor in the range of 0 to 1 [117]. Due to the existence of foreign surface, this lowers the nucleation barrier in the heterogeneous nucleation process. Therefore, it is crucial to understand the fundamentals of heterogeneous nucleation in order to achieve control over the nucleation process. In fact, as the foreign substances are embedded into water, they also affect the structure of interfacial water. To maximize the hydrogen bondings of water, the dissolved ions are expected to be accumulated at the surfaces of the substances. Due to the appearance of foreign substances, it is helpful to form the ion aggregate at the foreign surfaces, which may facilitate the phase transition (heterogeneous nucleation). Additionally, in combination with recent study [38], it is derived that heterogeneous nucleation may be affected by the geometric characteristics of foreign surface, especially geometric shape. In addition, it is also related to the molecular polarity of foreign surface. Further study is necessary.

### 4.2. Directional Natures of Hydrophobic Interactions

As the solutes are aggregated in aqueous solutions, it may be divided into H1w and H2s hydrophobic processes. In our recent study [37], different directional natures are found in the H1w and H2s processes. In the H1w process, the dissolved solutes are expected to approach in the specific direction with the lowest energy barrier, in which fewer expelled water molecules are expected. However, as the solutes come into contact in the H2s hydrophobic process (<R_H_), the solutes may be aggregated to minimize the ratio of surface area to volume of them. Additionally, the necessary pathway, where the solutes tend to pass through, may be expected while they are aggregated in water. Based on our recent study [37] on directional nature of hydrophobic interactions, it is utilized to understand the nature of molecular recognition, especially the molecular specificity.

Molecular recognition is closely related to biological process and drug design. In the process of molecular recognition, two or more binding partners may interact through non-covalent interactions to form a specific complex in solutions. Numerous works have been devoted to understanding the nature of molecular recognition. To date, three structural models have been proposed, such as Fischer’s lock-and-key model [118], Koshland’s induced fit model [119], and the conformational selection model [120,121,122,123]. Based on the lock-and-key model, it is assumed that the conformation of the free protein is the same as that of ligand-bound protein. In the induced fit model, the conformational differences are expected after the ligand is associated with the target protein. Therefore, a new conformation, which may be more complementary to the binding partner, is expected for the protein. According to the conformational selection model, this means that all protein conformations pre-exist, and the ligand may select the most favored conformation. After the ligand is bound with the protein, a population shift may be expected, which may lead to redistribution of the conformational states.

In the process of molecular recognition, it is necessary to require the molecular specificity and affinity. Molecular specificity may be defined as why two or more binding partners approach and bind together to form a specific complex in aqueous environment [124,125,126,127]. Regarding the binding affinity, it determines whether the complex will be formed in aqueous environments, which is related to the strength of non-covalent interactions between partners. In other words, the binding affinity may be closely related to the thermodynamic stability of the molecular association in aqueous solutions.

In fact, the molecules are rarely rigid. Therefore, the conformational changes may be expected for partners before and after they are associated in aqueous solutions. However, it is well known that shape complementarity plays an important role as a ligand is associated with a target protein. In fact, this is emphasized in each structural model of molecular recognition. This means that, when a ligand is associated with a given target protein, it must fit into the cavity of the protein without steric conflicts. Based on our studies [34,36,37] on hydrophobic interactions, this may agree with the minimization of the ratio of surface area to volume of the solutes in the H2s process.

**Figure 13:**
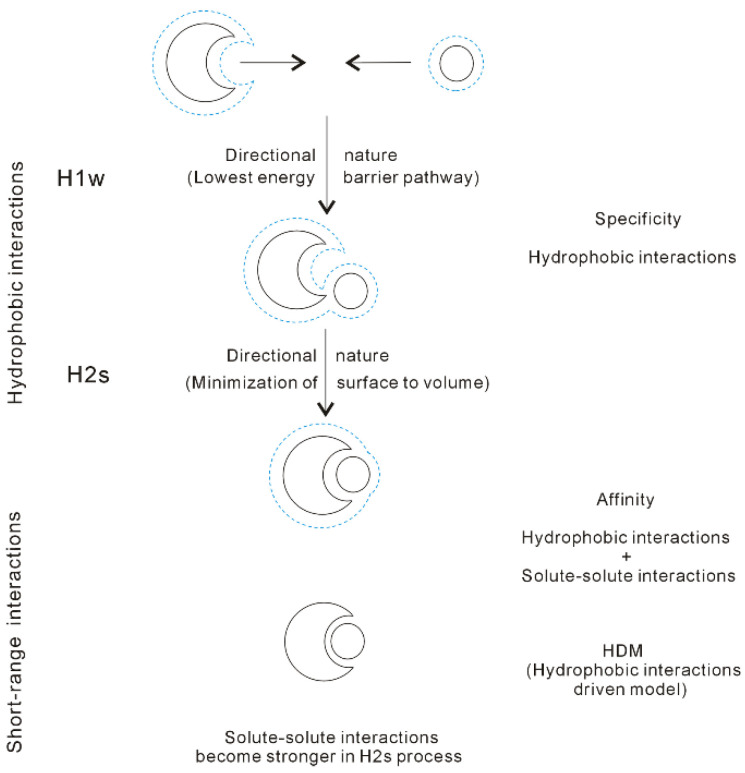
Hydrophobic-interaction-driven model (HDM) of molecular recognition. The solutes mainly affect the structure of interfacial water (dashed line). Due to hydrophobic interactions, the solutes are attracted and approach in the direction with the lowest energy barrier in the H1w process. As the solutes become contact in the H2s process, they are accumulated in a specific direction to minimize the surface area to volume ratio. Additionally, with decreasing separation between the solutes in the H2s process, the solute-solute interactions become stronger. The affinity of molecular recognition is related to both the hydrophobic interactions and the solute-solute interactions.

With decreasing the separation between partners, the direct interactions between them become stronger, such as van der Waals interactions, etc. In fact, it is necessary for partners to approach each other in solutions so that they are affected by the direct solute-solute interactions. Of course, this is due to hydrophobic interactions, which may lead to the dissolved solutes to approach each other in solutions. In other words, it seems that there exist the “attractive” forces between the solutes. Based on our recent work [37], molecular recognition is reasonably regarded to be driven by hydrophobic interactions (HDM, hydrophobic-interaction driven model [37]) (Figure 13).

From our recent studies [34,37], hydrophobic interactions may be closely related to both molecular specificity and affinity, especially the specificity of molecular recognition (Figure 13). According to HDM model, the molecular recognition may be divided into the following stages:

(I) Owing to the directional nature of hydrophobic interactions in H1w process, the dissolved solutes tend to approach in the specific direction with the lowest energy barrier, in which fewer water molecules between solutes may be expelled into bulk.

(II) When the solutes become contact in the H2s process, the solutes may be aggregated to minimize the ratio of surface area to volume of them. This is related to the directional nature of H2s hydrophobic process. In addition, this is utilized to explain the specificity in the process of molecular recognition.

(III) With decreasing distance between the partners, the direct intermolecular interactions between them become stronger, especially in the H2s process. Regarding the affinity of molecular recognition, it may be related to both hydrophobicity and the solute-solute interactions.

### 4.3. Temperature Effects on on Hydrophobic Interactions

**Figure 14:**
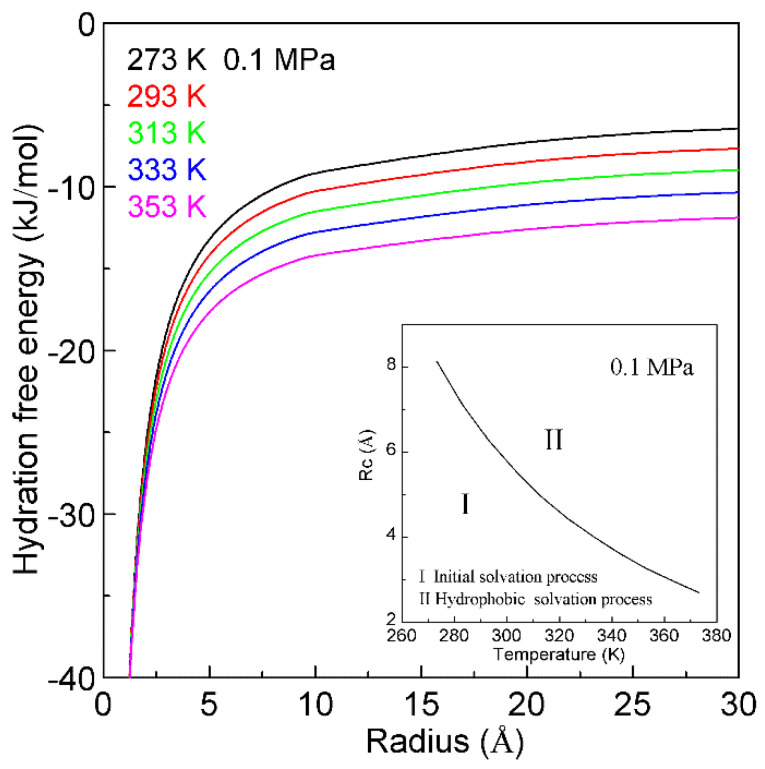
The temperature effects on hydration free energy at 0.1 MPa. Regarding the dependence of hydration free energy on temperature, it is related to the size of solute. With increasing temperature, this decreases the Rc.

Water plays an important role in the process of hydrophobic interactions. The hydrogen bondings of water may be influenced by temperature. Therefore, the changes of temperature undoubtedly affect the hydrophobic interactions. According to our recent study [128], the temperature effects on hydrophobic interactions may be related to the size of solute (Figure 14). With increasing temperature, this leads to the decrease of Rc (Figure 14). This may be utilized to investigate the nature of protein unfolding at high temperature (heat unfolding) and low temperature (cold denaturation).

In thermodynamics, proteins may be stable in a limited range of temperatures. Both low and high temperatures may make the native protein structure become unstable, which leads to the denaturation of protein structure. It is well known that the native structure of protein may be disrupted at high temperature. However, regarding the molecular mechanism of cold denaturation, it is still under debate [129,130,131,132,133,134]. To date, the cold denaturation is usually attributed to the decreasing strength of the hydrophobic interactions [129,130,131].

Hydrophobic interactions play a vital role in the process of protein folding. In general, protein folding may be regarded to be driven by hydrophobic interactions. Due to hydrophobic interactions, this leads to the structural collapse of nonpolar amino acids of protein in solutions, which decreases the separation between the residues of protein. During protein folding, the direct interactions between residues become stronger, such as van der Waals interactions, hydrogen bondings, etc. Additionally, hydrophobic interactions also play an important role to keep the native structure of protein stable. According to Pace et al. works [135,136], hydrophobic interactions may be treated as the dominant contribution to keep protein stable, which may be greater than 50%.

Thermodynamically, protein stability may be evaluated by the energy difference between the folded and unfolded state of the protein in aqueous solutions. This energy difference can be used to determine whether a protein will keep its native folded conformation at a low temperature (or a high temperature). Based on experimental measurements, the free energy difference between these states is usually between 20 and 60 kJ/mol [137]. Of course, this may be sufficient to prevent spontaneous unfolding at ambient conditions. However, it is noted that the protein native structure may be only marginally stable [138].

The changes of temperature may affect hydrophobic interactions, which undoubtedly affects the stability of protein structure. From our recent studies [34,35,36], hydrophobic interactions may be related to the size of solute. Therefore, it is necessary to understand the structural characteristics of protein. After the protein is globally regarded as a solute, the radii (R) are usually in the range from 12 and 23 Å [26], which is larger than Rc. Therefore, protein folding is wholly regarded to be driven by hydrophobic interactions. However, regarding the interior of protein, the average radius of amino acids is determined to be 3.3 Å with the standard deviation being 0.3 Å [26]. It is found that the average radius of amino acids (3.3 Å) is slightly larger than 3.25 Å, which is the Rc as two same solutes are embedded into ambient water [36]. Therefore, regarding the protein interior (local structure), it may be roughly engaged in the hydrophobic solvation process. Additionally, it is also engaged in initial solvation process. This is demonstrated by the buried water molecules, which are found in the interior of protein [139,140,141,142].

**Figure 15:**
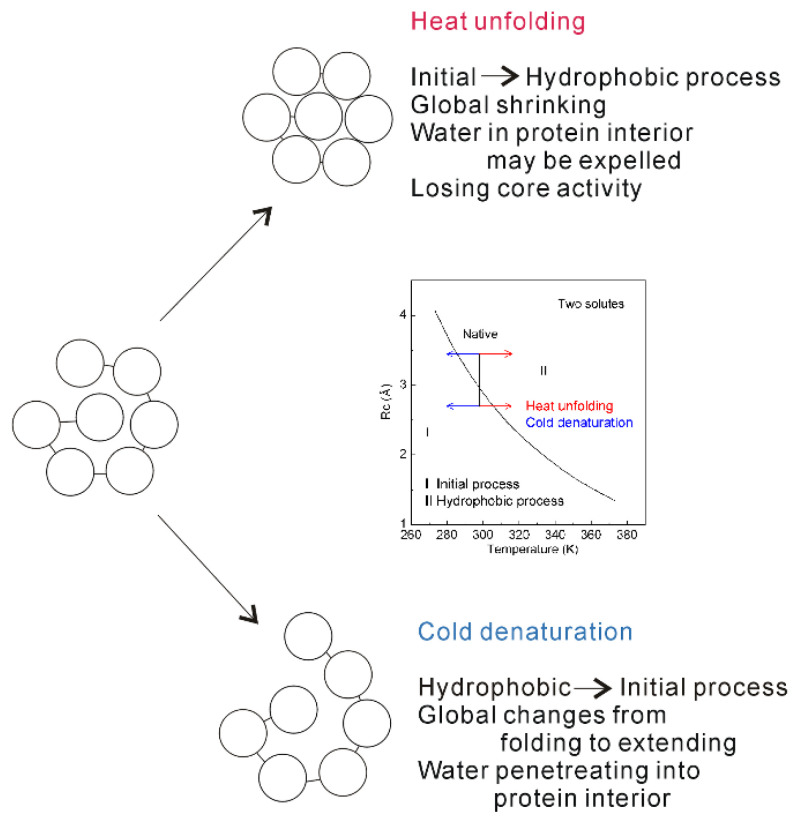
The mechanism of protein unfolding. Inlet shows the dependence of Rc on temperature as two identical solutes are embedded into water. With increasing (or decreasing) temperature, obvious structural changes are expected as the solute size passing through Rc. This is reflected on the global and local (interior) structures of protein. Regarding the molecular mechanism of protein unfolding, cold denaturation may be different from heat unfolding.

The dissolved behaviors of solutes in water may be related to the solute size. To understand the dependence of protein stability on temperature, it is necessary to investigate the changes of dissolved behaviors related to different solute size, being larger than Rc (>Rc), or less than Rc (<Rc). Temperature increase may lead to the decrease of Rc (Figure 15). With increasing temperature, the size of residue may change from being less than Rc to larger than Rc, and the obvious structural changes may be expected. In other words, the “repulsive” forces between solutes may be changed into the “attractive” forces. Therefore, with increasing temperature, the water molecules in the interior of protein (buried water) may be released into bulk water (Figure 15). Due to the expelled water, this may result in the accumulation in the interior of protein, and the global volume of protein is expected to become shrinking. Therefore, with increasing temperature, the inner structure of protein will be destroyed, which makes protein to lose the core activity.

Temperature decrease may lead to the increase of Rc (Figure 14). Therefore, protein stability is also affected by temperature decrease, which is different from the effects related to temperature increase. Due to temperature decrease, this may lead to the transition from hydrophobic to initial processes, which also affects the structure of protein (Figure 15). In other words, with decreasing temperature, water molecules may penetrate the core of protein (Figure 15). Therefore, the “repulsive” forces may be expected in the interior of protein. In addition, the global structure of protein may be changed from folded to extending conformation.

Regarding temperature effects on hydrophobic interactions, these are related to the size of solute. In combination with our recent studies [34,36,128], the obvious changes of dissolved behaviors of solutes are expected as the solute size passing though Rc. Therefore, the obvious structural difference may be expected between cold denaturation and heat unfolding. With decreasing temperature, this may lead to the transition from hydrophobic to initial process, water molecules are expected to penetrate the hydrophobic core of proteins [132] in cold denaturation, which leads to the increase of the solvent accessibility (surface area). Regarding heat unfolding, although global structure of protein will be preserved, this may result in the packing of the hydrophobic core during the transition from initial to hydrophobic processes. Therefore, cold denatured proteins are more expanded, and heat unfolded states become more compact [133,134]. Of course, these can be demonstrated by the experimental measurements [133,134]. From the above, cold denaturation may be different from heat unfolding.

Owing to hydrophobic interactions, the dissolved solutes may be aggregated in solutions in order to minimize their ratio of surface area to volume. Additionally, the dissolved solutes mainly affect the structure of interfacial water. Therefore, hydrophobic interactions may be related to the shape of solute. This may be applied to explain the molecular packing parameter. The parameter was proposed by Israelachvili, Mitchell, and Ninham [143], which was defined as v_0_/aI_0_, where v_0_ and I_0_ were the volume and the length of the surfactant tail and a was the surface area of the hydrophobic core of the aggregate expressed per molecule in the aggregate. The concept of molecular packing parameter is widely used in physics, chemistry, and biology because it allows a simple and intuitive insight into the self-assembly phenomenon. It is well known that there exists the following connection between the molecular packing parameter and the aggregate shape: 0 ≤ v_0_/aI_0_ ≤ 1/3 for sphere, 1/3 ≤ v_0_/aI_0_ ≤ 1/2 for cylinder, and 1/2 ≤ v_0_/aI_0_ ≤ 1 for bilayer [144]. Therefore, after the molecular packing parameter is determined, the shape and size of the equilibrium aggregate may be readily identified as shown above. From this work, it can be found that this parameter may be closely related to the effects of solute shape on hydrophobic interactions. Further study may be covered in our next work.

Hydrophobic interactions are involved in a lot of chemical and biological phenomena in aqueous environments. This review focuses on our current understanding on hydrophobic interactions, such as the dependence of hydrophobic interactions on the solute size, the directional natures of hydrophobic interactions, and the temperature effects on hydrophobic interactions. In fact, hydrophobic interactions may also be extended to investigate the molecular mechanism of protein folding, ion selectivity of nanopore (ion channel), Hofmeister effects, superhydrophobic surface, etc.

To maximize the hydrogen bondings of water, the dissolved solutes are attracted to approach each other until they may be affected by the direct solute-solute interactions. Therefore, hydrophobic interactions are reasonably regarded to be the fundamental driving force in the chemical and biological processes. Based on our recent study [34] on hydrophobic interactions, they are reasonably ascribed to the structural competition between interfacial and bulk water. Therefore, water plays a vital role in the process of hydrophobic effects. From our current understanding, water is reasonably regarded as “the director of a wonderful performance, not the audience”.

## 5. Conclusions

Hydrophobic interactions may be regarded as the fundamental driving force in numerous biophysical and biochemical processes in aqueous solutions. In this study, we focus on our current understanding on hydrophobic effects. Based on our recent studies, the following conclusions are derived:

(1) According to the structural studies on water and air-water interface, hydration free energy is determined, and applied to unravel the nature of hydrophobic effects. It is found that hydrophobic interactions may be attributed to the structural competition between interfacial and bulk water.

(2) Hydration free energy is related to the size of dissolved solute (or concentrations). With increasing the solute size, it may be divided into initial and hydrophobic solvation processes. In addition, various dissolved behaviors of solutes, such as dispersed and accumulated distributions in solutions, may be expected in initial and hydrophobic solvation processes. This is utilized to understand the origin of intermediate phase, which is found before solute nucleation occurs in solutions.

(3) While the dissolved solutes are accumulated in hydrophobic solvation process, it is divided into H1w and H2s hydrophobic processes. Additionally, different directional natures are expected in H1w and H2s processes. This is applied to understand the mechanism of molecular recognition, especially the specificity.

(4) Temperature changes may affect hydrophobic interactions, which may be related to solute size. With increasing temperature, this leads to the decrease of Rc. This is utilized to understand the nature of protein unfolding at high temperature (heat unfolding) and low temperature (cold denaturation). It can be found that cold denaturation may be different from heat unfolding.

## Data Availability

Not applicable.

## References

[1] Scheraga H.A. (1998). Theory of hydrophobic interactions. J. Biomol. Struct. Dyn..

[2] Blokzijl W., Engberts J.B.F.N. (1993). Hydrophobic effects. Opinions and facts. Angew. Chem. Int. Ed..

[3] Makowski M., Czaplewski C., Liwo A., Scheraga H.A. (2010). Potential of mean force of association of large hydrophobic particles: Toward the nanoscale limit. J. Phys. Chem. B.

[4] Sobolewski E., Makowski M., Czaplewski C., Liwo A., Ołdziej S., Scheraga H.A. (2007). Potential of mean force of hydrophobic association: Dependence on solute size. J. Phys. Chem. B.

[5] Bartosik A., Wiśniewska M., Makowski M. (2015). Potentials of mean force for hydrophobic interactions between hydrocarbons in water solution: Dependence on temperature, solute shape, and solute size. J. Phys. Org. Chem..

[6] Hansch C., Fujita T. (1964). p-σ-π analysis. A method for the correlation of biological activity and chemical structure. J. Am. Chem. Soc..

[7] Kujawski J., Popielarska H., Myka A., Drabińska B., Bernard M.K. (2012). The log P parameter as a molecular descriptor in the computer-aided drug design-an overview. Comput. Meth. Sci. Technol..

[8] Frank H.S., Evans M.W. (1945). Free volume and entropy in condensed systems III. Entropy in binary liquid mixtures; partial molal entropy in dilute solutions; structure and thermodynamics in aqueous electrolytes. J. Chem. Phys..

[9] Davis J.G., Gierszal K.P., Wang P., Ben-Amotz D. (2012). Water structural transformation at molecular hydrophobic interfaces. Nature.

[10] Galamba N. (2013). Water’s structure around hydrophobic solutes and the iceberg model. J. Phys. Chem. B.

[11] Raschke T.M., Levitt M. (2005). Nonpolar solutes enhance water structure within hydration shells while reducing interactions between them. Proc. Natl. Acad. Sci. USA.

[12] Rezus Y.L.A., Bakker H.J. (2007). Observation of immobilized water molecules around hydrophobic groups. Phys. Rev. Lett..

[13] Turner J., Soper A.K., Finney J.L. (1990). A neutron-diffraction study of tetramethylammonium chloride in aqueous solution. Mol. Phys..

[14] Qvist J., Halle B. (2008). Thermal signature of hydrophobic hydration dynamics. J. Am. Chem. Soc..

[15] Buchanan P., Aldiwan N., Soper A.K., Creek J.L., Koh C.A. (2005). Decreased structure on dissolving methane in water. Chem. Phys. Lett..

[16] Bakulin A.A., Liang C., Jansen T.L., Wiersma D.A., Bakker H.J., Pshenichnikov M.S. (2009). Hydrophobic solvation: A 2D IR spectroscopic inquest. Acc. Chem. Res..

[17] Sun Q. (2020). The effects of dissolved hydrophobic and hydrophilic groups on water structure. J. Solution Chem..

[18] Kauzmann W. (1959). Some factors in the interpretation of protein denaturation. Adv. Protein Chem..

[19] Ferguson S.B., Seward E.M., Diederich F., Sanford E.M., Chou A., Inocencio-Szweda P., Knobler C.B. (1988). Strong enthalpically driven complexation of neutral benzene guests in aqueous solution. J. Org. Chem..

[20] Baron R., Setny P., McCammon J.A. (2010). Water in cavity-ligand recognition. J. Am. Chem. Soc..

[21] Setny P., Baron R., McCammon J.A. (2010). How can hydrophobic association be enthalpy driven?. J. Chem. Theory Comput..

[22] Hummer G. (2010). Molecular binding under water’s influence. Nat. Chem..

[23] Huang D.M., Geissler P.L., Chandler D. (2001). Scaling of hydrophobic solvation free energies. J. Phys. Chem. B.

[24] Lum K., Chandler D., Weeks J.D. (1999). Hydrophobicity at small and large length scales. J. Phys. Chem. B.

[25] Chandler D. (2005). Interfaces and the driving force of hydrophobic assembly. Nature.

[26] Huang D.M., Chandler D. (2000). Temperature and length scale dependence of hydrophobic effects and their possible implications for protein folding. Proc. Natl. Acad. Sci. USA.

[27] Ball P. (2008). Water as an active constituent in cell biology. Chem. Rev..

[28] Ball P. (2017). Water is an active matrix of life for cell and molecular biology. Proc. Natl. Acad. Sci. USA.

[29] Ball P. (2011). More than a bystander. Nature.

[30] Sun Q. (2009). The Raman OH stretching bands of liquid water. Vib. Spectrosc..

[31] Sun Q. (2012). Raman spectroscopic study of the effects of dissolved NaCl on water structure. Vib. Spectrosc..

[32] Sun Q. (2013). Local statistical interpretation for water structure. Chem. Phys. Lett..

[33] Sun Q., Guo Y. (2016). Vibrational sum frequency generation spectroscopy of the air/water interface. J. Mol. Liq..

[34] Sun Q. (2017). The physical origin of hydrophobic effects. Chem. Phys. Lett..

[35] Sun Q., Zhang M.X., Cui S. (2019). The structural origin of hydration repulsive force. Chem. Phys. Lett..

[36] Sun Q., Su X.W., Cheng C.B. (2019). The dependence of hydrophobic interactions on the solute size. Chem. Phys..

[37] Sun Q., Wang W.Q., Cui S. (2021). Directional nature of hydrophobic interactions: Implications for the mechanism of molecular recognition. Chem. Phys..

[38] Sun Q., Cui S., Zhang M.X. (2020). Homogeneous nucleation mechanism of NaCl in aqueous solutions. Crystals.

[39] Stanley H.E., Teixeira J. (1980). Interpretation of the unusual behavior of H_2_O and D_2_O at low temperatures: Tests of a percolation model. J. Chem. Phys..

[40] Nilsson A., Pettersson L.G.M. (2011). Perspective on the structure of liquid water. Chem. Phys..

[41] Röntgen W.C. (1892). Ueber die Constitution des flüssigen Wassers. Ann. Phys..

[42] Russo J., Tanaka H. (2014). Understanding water’s anomalies with locally favoured structures. Nat. Commun..

[43] Hamm P. (2016). Markov state model of the two-state behaviour of water. J. Chem. Phys..

[44] Shi R., Tanaka H. (2018). Microscopic structural descriptor of liquid water. J. Chem. Phys..

[45] Skinner L.B., Huang C., Schlesinger D., Pettersson L.G.M., Nilsson A., Benmore C.J. (2013). Benchmark oxygen-oxygen pair-distribution function of ambient water from X-ray diffraction measurements with a wide Q-range. J. Chem. Phys..

[46] Hura G., Sorenson J.M., Glaeser R.M., Head-Gordon T. (2000). A high-quality X-ray scattering experiment on liquid water at ambient conditions. J. Chem. Phys..

[47] Misquitta A.J., Szalewicz K. (2002). Intermolecular forces from asymptotically corrected density functional description of monomers. Chem. Phys. Lett..

[48] Misquitta A.J., Jeziorski B., Szalewicz K. (2003). Dispersion energy from density-functional theory description of monomers. Phys. Rev. Lett..

[49] Hoja J., Sax A.F., Szalewicz K. (2014). Is electrostatics sufficient to describe hydrogen-bonding interactions?. Chem. Eur. J..

[50] Fraley P.E., Rao K.N. (1969). High resolution infrared spectra of water vapor: ν_1_ and ν_3_ band of H_2_^16^O. J. Mol. Spectrosc..

[51] Ludwig R. (2002). The effect of hydrogen bonding on the thermodynamic and spectroscopic properties of molecular clusters and liquids. Phys. Chem. Chem. Phys..

[52] Smith J.D., Cappa C.D., Wilson K.R., Cohen R.C., Geissler P.L., Saykally R.J. (2005). Unified description of temperature-dependent hydrogen-bond rearrangements in liquid water. Proc. Natl. Acad. Sci. USA.

[53] Huang C., Wikfeldt K.T., Tokushima T., Nordlund D., Harada Y., Bergmann U., Niebuhr M., Weiss T.M., Horikawa Y., Leetmaa M. (2009). The inhomogeneous structure of water at ambient conditions. Proc. Natl. Acad. Sci. USA.

[54] Kim K.H., Späh A., Pathak H., Perakis F., Mariedahl D., Amann-Winkel K., Sellberg J.A., Lee J.H., Kim S., Park J. (2017). Maxima in the thermodynamic response and correlation functions of deeply supercooled water. Science.

[55] Matsumoto M., Saito S., Ohmine I. (2002). Molecular dynamics simulation of the ice nucleation and growth process leading to water freezing. Nature.

[56] Moore E.B., Molinero V. (2011). Structural transformation in supercooled water controls the crystallization rate of ice. Nature.

[57] Fitzner M., Sosso G.C., Cox S.J., Michaelides A. (2019). Ice is born in low-mobility regions of supercooled liquid water. Proc. Natl. Acad. Sci. USA.

[58] Trudu F., Donadio D., Parrinello M. (2006). Freezing of a Lennard-Jones fluid: From nucleation to spinodal regime. Phys. Rev. Lett..

[59] Berryman J.T., Anwar M., Dorosz S., Schilling T. (2016). The early crystal nucleation process in hard spheres shows synchronised ordering and densification. J. Chem. Phys..

[60] Desgranges C., Delhommelle J. (2019). Can ordered precursors promote the nucleation of solid solutions?. Phys. Rev. Lett..

[61] Speedy R.J. (1982). Limiting forms of the thermodynamic divergences at the conjectured stability limits in superheated and supercooled water. J. Phys. Chem..

[62] Poole P.H., Sciortino F., Essmann U., Stanley H.E. (1992). Phase behaviour of metastable water. Nature.

[63] Sastry S., Debenedetti P.G., Sciortino F., Stanley H.E. (1996). Singularity-free interpretation of the thermodynamics of supercooled water. Phys. Rev. E.

[64] Angell C.A. (2008). Insights into phases of liquid water from study of its unusual glass-forming properties. Science.

[65] Kim K.H., Amann-Winkel K., Giovambattista N., Späh A., Perakis F., Pathak H., Parada M.L., Yang C., Mariedahl D., Eklund T. (2020). Experimental observation of the liquid-liquid transition in bulk supercooled water under pressure. Science.

[66] Palmer J.C., Martelli F., Liu Y., Car R., Panagiotopoulos A.Z., Debenedetti P.G. (2014). Metastable liquid-liquid transition in a molecular model of water. Nature.

[67] Debenedetti P.G., Sciortino F., Zerze G.H. (2020). Second critical point in two realistic models of water. Science.

[68] Gartner T.E., Zhang L., Piaggi P.M., Car R., Panagiotopoulos A.Z., Debenedetti P.G. (2020). Signatures of a liquid-liquid transition in an ab initio deep neural network model for water. Proc. Natl. Acad. Sci. USA.

[69] Handle P.H., Loerting T., Sciortino F. (2017). Supercooled and glassy water: Metastable liquid(s), amorphous solid(s), and a no-man’s land. Proc. Natl. Acad. Sci. USA.

[70] Collins K.D., Neilson G.W., Enderby J.E. (2007). Ions in water: Characterizing the forces that control chemical processes and biological structure. Biophys. Chem..

[71] Cappa C.D., Smith J.D., Messer B.M., Cohen R.C., Saykally R.J. (2006). Effects of cations on the hydrogen bond network of liquid water: New results from X-ray absorption spectroscopy of liquid microjets. J. Phys. Chem. B.

[72] Omta A.W., Kropman M.F., Woutersen S., Bakker H.J. (2003). Negligible effect of ions on the hydrogen-bond structure in liquid water. Science.

[73] Moilanen D.E., Wong D., Rosenfeld D.E., Fenn E.E., Fayer M.D. (2009). Ion-water hydrogen-bond switching observed with 2D IR vibrational echo chemical exchange spectroscopy. Proc. Natl. Acad. Sci. USA.

[74] Turton D.A., Hunger J., Hefter G., Buchner R., Wynne K. (2008). Glasslike behavior in aqueous electrolyte solutions. J. Chem. Phys..

[75] Sun Q. (2010). The single donor-single acceptor hydrogen bonding structure in water probed by Raman spectroscopy. J. Chem. Phys..

[76] Scatena L.F., Brown M.G., Richmond G.L. (2001). Water at hydrophobic surfaces: Weak hydrogen bonding and strong orientation effects. Science.

[77] Jubb A.M., Hua W., Allen H.C. (2012). Organization of water and atmospherically relevant ions and solutes: Vibrational sum frequency spectroscopy at the vapor/liquid and liquid/solid interfaces. Acc. Chem. Res..

[78] Shen Y.R., Ostroverkhov V. (2006). Sum-frequency vibrational spectroscopy on water interfaces: Polar orientation of water molecules at interfaces. Chem. Rev..

[79] Richmond G.L. (2002). Molecular bonding and interactions at aqueous surfaces as probed by vibrational sum frequency spectroscopy. Chem. Rev..

[80] Tian C.S., Shen Y.R. (2009). Sum-frequency vibrational spectroscopic studies of water/vapor interfaces. Chem. Phys. Lett..

[81] Ji N., Ostroverkhov V., Tian C.S., Shen Y.R. (2008). Characterization of vibrational resonances of water-vapor interfaces by phase-sensitive sum-frequency spectroscopy. Phys. Rev. Lett..

[82] Thompson H., Soper A.K., Ricci M.A., Bruni F., Skipper N.T. (2007). The three-dimensional structure of water confined in nanoporous vycor glass. J. Phys. Chem. B.

[83] Giri A.K., Teixeira F., Cordeiro M.N.D.S. (2018). Structure and kinetics of water in highly confined conditions: A molecular dynamics simulation study. J. Mol. Liq..

[84] Winarto, Takaiwa D., Yamamoto E., Yasuoka K. (2015). Structures of water molecules in carbon nanotubes under electric fields. J. Chem. Phys..

[85] Dorsey N.E. (1940). Properties of Ordinary Water Substance.

[86] Ashbaugh H.S., Weiss K., Williams S.M., Meng B., Surampudi L.N. (2015). Temperature and pressure dependence of methane correlations and osmotic second virial coefficients in water. J. Phys. Chem. B.

[87] Chodera J.D., Mobley D.L. (2013). Entropy-enthalpy compensation: Role and ramifications in biomolecular ligand recognition and design. Annu. Rev. Biophys..

[88] Dragan A.I., Read C.M., Crane-Robinson C. (2017). Enthalpy-entropy compensation: The role of solvation. Eur. Biophys. J..

[89] Breiten B., Lockett M.R., Sherman W., Fujita S., Al-Sayah M., Lange H., Bowers C.M., Heroux A., Krilov G., Whitesides G.M. (2013). Water networks contribute to enthalpy/entropy compensation in protein-ligand binding. J. Am. Chem. Soc..

[90] Sharp K. (2001). Entropy-enthalpy compensation: Fact or artifact?. Protein Sci..

[91] Gilli P., Ferrett V., Gilli G., Borea P.A. (1994). Enthalpy-entropy compensation in drug-receptor binding. J. Phys. Chem..

[92] Zangi R. (2011). Driving force for hydrophobic interaction at different length scales. J. Phys. Chem. B.

[93] Uddin N.M., Capaldi F.M., Farouk B. (2012). Molecular dynamics simulations of carbon nanotube dispersions in water: Effects of nanotube length, diameter, chirality and surfactant structures. Comput. Mat. Sci..

[94] Li L.W., Bedrov D., Smith G.D. (2006). Water-induced interactions between carbon nanoparticles. J. Phys. Chem. B.

[95] Wallqvist A., Berne B.J. (1995). Computer simulation of hydrophobic hydration forces on stacked plates at short range. J. Phys. Chem..

[96] Wallqvist A., Berne B.J. (1995). Molecular dynamics study of the dependence of water solvation free energy on solute curvature and surface area. J. Phys. Chem..

[97] Hummer G., Rasaiah J.C., Noworyta J.P. (2001). Water conduction through the hydrophobic channel of a carbon nanotube. Nature.

[98] Giovanbattista N., Debenedetti P.G., Rossky P.J. (2007). Hydration behavior under confinement by nanoscale surfaces with patterned hydrophobicity and hydrophilicity. J. Phys. Chem. C.

[99] Liu P., Huang X., Zhou R., Berne B.J. (2005). Observation of a dewetting transition in the collapse of the melittin tetramer. Nature.

[100] ten Wolde P.R., Chandler D. (2002). Drying induced hydrophobic polymer collapse. Proc. Natl. Acad. Sci. USA.

[101] Khouzani M.F., Chevrier D.M., Güttlein P., Hauser K., Zhang P., Hedinc N., Gebauer D. (2015). Disordered amorphous calcium carbonate from direct precipitation. Cryst. Eng. Comm..

[102] Pouget E.M., Bomans P.H.H., Goos J.A.C.M., Frederik P.M., de With G., Sommerdijk N.A.J.M. (2009). The initial stages of template-controlled CaCO_3_ formation revealed by cryo-TEM. Science.

[103] ten Wolde P.R., Frenkel D. (1997). Enhancement of protein crystal nucleation by critical density fluctuations. Science.

[104] Gebauer D., Colfen H. (2011). Prenucleation clusters and non-classical nucleation. Nano Today.

[105] Gebauer D., Kellermeier M., Gale J.D., Bergstrom L., Colfen H. (2014). Pre-nucleation clusters as solute precursors in crystallization. Chem. Soc. Rev..

[106] Vekilov P.G. (2010). The two-step mechanism of nucleation of crystals in solution. Nanoscale.

[107] Karthika S., Radhakrishnan T.K., Kalaichelvi P. (2016). A review of classical and nonclassical nucleation theories. Cryst. Growth Des..

[108] Giberti F., Tribello G.A., Parrinello M. (2013). Transient polymorphism in NaCl. J. Chem. Theory Comput..

[109] Zimmermann N.E.R., Vorselaars B., Quigley D., Peters B. (2015). Nucleation of NaCl from aqueous solution: Critical sizes, ion-attachment kinetics, and rates. J. Am. Chem. Soc..

[110] Alejandre J., Hansen J.P. (2007). Ions in water: From ion clustering to crystal nucleation. Phys. Rev. E.

[111] Chakraborty D., Patey G.N. (2013). How crystals nucleate and grow in aqueous NaCl solution. J. Phys. Chem. Lett..

[112] Lanaro G., Patey G.N. (2016). Birth of NaCl crystals: Insights from molecular simulations. J. Phys. Chem. B.

[113] Chakraborty D., Patey G.N. (2013). Evidence that crystal nucleation in aqueous NaCl solution occurs by the two-step mechanism. Chem. Phys. Lett..

[114] Jiang H., Debenedetti P.G., Panagiotopoulos A.Z. (2019). Nucleation in aqueous NaCl solutions shifts from 1-step to 2-step mechanism on crossing the spinodal. J. Chem. Phys..

[115] Patel L.A., Kindt J.T. (2019). Simulations of NaCl aggregation from solution: Solvent determines topography of free energy landscape. J. Comput. Chem..

[116] Sosso G.C., Chen J., Cox S.J., Fitzner M., Pedevilla P., Zen A., Michaelides A. (2016). Crystal nucleation in liquids: Open questions and future challenges in molecular dynamics simulations. Chem. Rev..

[117] Kalikmanov V. (2013). Nucleation Theory.

[118] Fischer E. (1894). Einfluss der configuration auf die wirkung der enzyme. Ber. Dtsch. Chem. Ges..

[119] Koshland D.E.J. (1958). Application of a theory of enzyme specificity to protein synthesis. Proc. Natl. Acad. Sci. USA.

[120] Ma B., Kumar S., Tsai C.J., Nussinov R. (1999). Folding funnels and binding mechanisms. Protein Eng..

[121] Tsai C.J., Kumar S., Ma B., Nussinov R. (1999). Folding funnels, binding funnels, and protein function. Protein Sci..

[122] Tobi D., Bahar I. (2005). Structural changes involved in protein binding correlate with intrinsic motions of proteins in the unbound state. Proc. Natl. Acad. Sci. USA.

[123] Csermely P., Palotai R., Nussinov R. (2010). Induced fit, conformational selection and independent dynamic segments: An extended view of binding events. Trends Biochem. Sci..

[124] Janin J. (1995). Principles of protein-protein recognition from structure to thermodynamics. Biochimie.

[125] Havranek J.J., Harbury P.B. (2003). Automated design of specificity in molecular recognition. Nat. Struct. Biol..

[126] Kortemme T., Joachimiak L.A., Bullock A.N., Schuler A.D., Stoddard B.L., Baker D. (2004). Computational redesign of protein-protein interaction specificity. Nat. Struct. Biol..

[127] Bolon D.N., Grant R.A., Baker T.A., Sauer R.T. (2005). Specificity versus stability in computational protein design. Proc. Natl. Acad. Sci. USA.

[128] Sun Q., Fu Y.F., Wang W.Q. (2022). Temperature effects on hydrophobic interactions: Implications for protein unfolding. Chem. Phys..

[129] Dias C.L. (2012). Unifying microscopic mechanism for pressure and cold denaturations of proteins. Phys. Rev. Lett..

[130] Dias C.L., Ala-Nissila T., Wong-ekkabut J., Vattulainen I., Grant M., Karttunen M. (2010). The hydrophobic effect and its role in cold denaturation. Cryobiology.

[131] Dias C.L., Ala-Nissila T., Karttunen M., Vattulainen I., Grant M. (2008). Microscopic mechanism for cold denaturation. Phys. Rev. Lett..

[132] Ramirez-Sarmiento C.A., Baez M., Wilson C.A.M., Babul J., Komives E.A., Guixe V. (2013). Observation of solvent penetration during cold denaturation of E. coli phosphofructokinase-2. Biophys. J..

[133] Nettels D., Müller-Späth S., Küster F., Hofmann H., Haenni D., Rüegger S., Reymond L., Hoffmann A., Kubelka J., Heinz B. (2009). Single-molecule spectroscopy of the temperature-induced collapse of unfolded proteins. Proc. Natl. Acad. Sci. USA.

[134] Camilloni C., Bonetti D., Morrone A., Giri R., Dobson C.M., Brunori M., Gianni S., Vendruscolo M. (2016). Towards a structural biology of the hydrophobic effect in protein Folding. Sci. Rep..

[135] Pace C.N., Fu H., Fryar K.L., Landua J., Trevino S.R., Shirley B.A., Hendricks M.M., Iimura S., Gajiwala K., Scholtz J.M. (2011). Contribution of hydrophobic interactions to protein stability. J. Mol. Biol..

[136] Pace C.N., Scholtz J.M., Grimsley G.R. (2014). Forces stabilizing proteins. FEBS Lett..

[137] Gething M.J., Sambrook J. (1992). Protein folding in the cell. Nature.

[138] Dill K.A. (1985). Theory for the folding and stability of globular proteins. Biochemistry.

[139] Levitt M., Park B.H. (1993). Water: Now you see it, now you don’t. Structure.

[140] Schoenborn B.P., Garcia A., Knott R. (1995). Hydration in protein crystallography. Prog. Biophys. Mol. Biol..

[141] Williams M.A., Goodfellow J.M., Thornton J.M. (1994). Buried waters and internal cavities in monomeric proteins. Protein Sci..

[142] Carugo O. (2016). Statistical survey of the buried waters in the Protein Data Bank. Amino Acids..

[143] Israelachvili J.N., Mitchell D.J., Ninham B.W. (1976). Theory of self-assembly of hydrocarbon amphiphiles into micelles and bilayers. J. Chem. Soc. Faraday Trans..

[144] Nagarajan R. (2002). Molecular packing parameter and surfactant self-assembly: The neglected role of the surfactant tail. Langmuir.

